# Landscapes of binding antibody and T-cell responses to pox-protein HIV vaccines in Thais and South Africans

**DOI:** 10.1371/journal.pone.0226803

**Published:** 2020-01-30

**Authors:** Lue Ping Zhao, Andrew Fiore-Gartland, Lindsay N. Carpp, Kristen W. Cohen, Nadine Rouphael, Llewellyn Fleurs, One Dintwe, Michael Zhao, Zoe Moodie, Youyi Fong, Nigel Garrett, Ying Huang, Craig Innes, Holly E. Janes, Erica Lazarus, Nelson L. Michael, Sorachai Nitayaphan, Punnee Pitisuttithum, Supachai Rerks-Ngarm, Merlin L. Robb, Stephen C. De Rosa, Lawrence Corey, Glenda E. Gray, Kelly E. Seaton, Nicole L. Yates, M. Juliana McElrath, Nicole Frahm, Georgia D. Tomaras, Peter B. Gilbert

**Affiliations:** 1 Division of Public Health Sciences, Fred Hutchinson Cancer Research Center, Seattle, Washington, United States of America; 2 Vaccine and Infectious Disease Division, Fred Hutchinson Cancer Research Center, Seattle, Washington, United States of America; 3 Hope Clinic of the Emory Vaccine Center, Division of Infectious Diseases, Emory University, Atlanta, Georgia, United States of America; 4 Desmond Tutu HIV Centre, University of Cape Town, Cape Town, South Africa; 5 Cape Town HVTN Immunology Laboratory, Hutchinson Centre Research Institute of South Africa, NPC (HCRISA), Cape Town, South Africa; 6 Department of Informatics, School of Arts and Sciences, University of Washington, Seattle, Washington, United States of America; 7 Centre for the AIDS Programme of Research in South Africa (CAPRISA), University of KwaZulu-Natal, Durban, South Africa; 8 The Aurum Institute, Klerksdorp Research Centre, Klerksdorp, South Africa; 9 Perinatal HIV Research Unit, Faculty of Health Sciences, University of the Witwatersrand, Johannesburg, South Africa; 10 U.S. Military HIV Research Program, Walter Reed Army Institute of Research, Silver Spring, Maryland, United States of America; 11 Henry M. Jackson Foundation for the Advancement of Military Medicine, Bethesda, Maryland, United States of America; 12 Armed Forces Research Institute of Medical Sciences (AFRIMS), Bangkok, Thailand; 13 Vaccine Trial Centre, Faculty of Tropical Medicine, Mahidol University, Bangkok, Thailand; 14 Department of Disease Control, C/O Ministry of Public Health, Nonthaburi, Thailand; 15 South African Medical Research Council, Cape Town, South Africa; 16 Duke Human Vaccine Institute, Duke University School of Medicine, Durham, North Carolina, United States of America; 17 Department of Medicine, Duke University School of Medicine, Durham, North Carolina, United States of America; 18 Bill & Melinda Gates Medical Research Institute, Cambridge, Massachusetts, United States of America; 19 Department of Surgery, Duke University, Durham, North Carolina, United States of America; Uniformed Services University, UNITED STATES

## Abstract

**Background:**

HIV vaccine trials routinely measure multiple vaccine-elicited immune responses to compare regimens and study their potential associations with protection. Here we employ unsupervised learning tools facilitated by a bidirectional power transformation to explore the multivariate binding antibody and T-cell response patterns of immune responses elicited by two pox-protein HIV vaccine regimens. Both regimens utilized a recombinant canarypox vector (ALVAC-HIV) prime and a bivalent recombinant HIV-1 Envelope glycoprotein 120 subunit boost. We hypothesized that within each trial, there were participant subgroups sharing similar immune responses and that their frequencies differed across trials.

**Methods and findings:**

We analyzed data from three trials–RV144 (NCT00223080), HVTN 097 (NCT02109354), and HVTN 100 (NCT02404311), the latter of which was pivotal in advancing the tested pox-protein HIV vaccine regimen to the HVTN 702 Phase 2b/3 efficacy trial. We found that bivariate CD4+ T-cell and anti-V1V2 IgG/IgG3 antibody response patterns were similar by age, sex-at-birth, and body mass index, but differed for the pox-protein clade AE/B alum-adjuvanted regimen studied in RV144 and HVTN 097 (P_AE/B_/alum) compared to the pox-protein clade C/C MF59-adjuvanted regimen studied in HVTN 100 (P_C_/MF59). Specifically, more P_AE/B_/alum recipients had low CD4+ T-cell and high anti-V1V2 IgG/IgG3 responses, and more P_C_/MF59 recipients had broad responses of both types. Analyses limited to “vaccine-matched” antigens suggested that some of the differences in responses between the regimens could have been due to antigens in the assays that did not match the vaccine immunogens. Our approach was also useful in identifying subgroups with unusually absent or high co-responses across assay types, flagging individuals for further characterization by functional assays. We also found that co-responses of anti-V1V2 IgG/IgG3 and CD4+ T cells had broad variability. As additional immune response assays are standardized and validated, we anticipate our framework will be increasingly valuable for multivariate analysis.

**Conclusions:**

Our approach can be used to advance vaccine development objectives, including the characterization and comparison of candidate vaccine multivariate immune responses and improved design of studies to identify correlates of protection. For instance, results suggested that HVTN 702 will have adequate power to interrogate immune correlates involving anti-V1V2 IgG/IgG3 and CD4+ T-cell co-readouts, but will have lower power to study anti-gp120/gp140 IgG/IgG3 due to their lower dynamic ranges. The findings also generate hypotheses for future testing in experimental and computational analyses aimed at achieving a mechanistic understanding of vaccine-elicited immune response heterogeneity.

## Introduction

The current global HIV incidence-to-prevalence ratio of 0.05 indicates that without more effective prevention tools the total number of people living with HIV globally will continue to increase [1]. The quest to design a safe and effective preventative HIV vaccine, which is believed to be a critical tool for controlling the current HIV pandemic [2, 3], has been hindered by pathogen variability and immune escape, a lack of knowledge of immune correlates of protection, and an incomplete understanding of the variation in vaccine-induced immune responses [4]. New quantitative approaches may help to tackle these pressing problems.

Out of the six phase 3 preventative HIV vaccine efficacy trials that have been performed to date [5–10], only the RV144 trial of a recombinant canarypox vector vaccine (ALVAC-HIV of clade AE) and a bivalent recombinant HIV-1 Envelope (Env) glycoprotein 120 (gp120) subunit vaccine (AIDSVAX B/E) prime-boost regimen (hereafter referred to as the P_AE/B_/alum regimen, where “P” stands for “pox-protein”), conducted in an HIV-seronegative population in Thailand where HIV subtype CRF01_AE B/E is dominant [11, 12], demonstrated modest vaccine efficacy [9]. Although the estimated efficacy was 31% at Month 42, the RV144 trial nevertheless provided the first evidence that vaccination can prevent HIV acquisition [9]. Immune correlates analyses of RV144 vaccine recipients revealed that IgG binding antibody responses to the HIV Env V1V2 loop were inversely correlated with risk of HIV infection [13, 14], whereas certain Env-specific IgA antibody responses were positively correlated with risk of HIV infection [13, 15]. Subsequent analyses identified additional correlates of risk, including polyfunctionality of Env-specific CD4+ T-cell responses [16–18]. Further analyses also suggested that host factors such as immunogenetics, body mass index (BMI), and other demographic variables influence immune responses to HIV vaccination and/or vaccine efficacy [19–24]. The HIV Vaccine Trials Network (HVTN) has been designing and conducting studies to build upon these results, as detailed below.

In the HVTN 097 trial, the P_AE/B_/alum regimen tested in the RV144 trial was administered to participants in South Africa, where HIV subtype C, as opposed to subtype AE, predominates. Cellular and humoral responses previously found to correlate with risk of HIV infection in the RV144 trial were then assessed, as were potential associations of race/ethnicity, sex-at-birth, and age with vaccine-matched and cross-clade responses [25]. A major conclusion from the HVTN 097 trial was that immune responses found to inversely correlate with risk in the RV144 trial were elicited at similar (or sometimes greater) rates/magnitudes in a South African population compared to the RV144 Thai population.

For the HVTN 100 trial, which was also performed in South Africa, the P_AE/B_/alum regimen was regionally-adapted to use a subtype C gp120 in the ALVAC vector and two subtype C recombinant gp120s (ALVAC-gp120_C_), the alum adjuvant was changed to MF59, and an additional boost was added in the immunization series. This vaccine regimen, hereafter referred to as the P_C_/MF59 regimen, represented the first effort to modify and potentially improve upon the original P_AE/B_/alum regimen [26]. Analysis of the HVTN 100 results revealed that the P_C_/MF59 regimen elicited humoral and cellular responses that were associated with reduced risk of HIV acquisition in RV144, although with different response patterns, as further described here. All four of the pre-specified criteria for advancement of the P_C_/MF59 regimen into a preventative efficacy trial were met, leading to the launch of the ongoing HVTN 702 phase 2b/3 efficacy trial of P_C_/MF59 versus placebo in South African men and women at risk of HIV acquisition.

Comparisons of vaccine-elicited immune responses within and across trials are important. Technological advancements have enabled increasingly large numbers of immune responses to be assessed in vaccine trials, raising the question of how best to compare multiple vaccine-induced responses across trials. One successful approach has been to synthesize several immune responses into score-type variables and to assess their associations with HIV acquisition [10, 13, 27]. In an alternative approach, Chung and colleagues used unsupervised learning techniques (hierarchical clustering and principal component analysis) to analyze six antibody Fc effector functions and 58 related biophysical measurements and identify vaccine-specific humoral signatures, finding further evidence to support IgG V1V2-targeting responses in protective immunity [18, 28]. Buoyed by these successes, we reasoned that developing additional multivariate approaches to globally compare immune response profiles across trials could help advance vaccine development objectives, including: A) to characterize and compare immunogenicity across vaccine regimens and other participant factors at a greater multivariate resolution than many previous analyses; B) to identify unusual vaccine response profiles of interest that could be missed by lower-dimensional analyses; C) to aid the design of immune correlates of risk and protection studies; D) to aid the interpretation of correlates of risk and protection analysis results; and E) to guide the selection of assays for future vaccine trials.

Toward these stated goals, we describe an unsupervised learning framework for analyzing a large number of immune responses measured from one or more trials and apply this framework to compare the immune response profiles of the pox protein HIV vaccine regimens studied in the RV144, HVTN 097, and HVTN 100 trials. A key element in this framework is the use of a bidirectional power transformation (BDPT) that transforms quantitative immune response measurements with a distinctively bimodal distribution, which increases the segregation of participants into responder versus non-responder categories. Further, BDPT-transformed immune response readouts within an assay are on a comparable quantitative scale, enabling direct comparison of intra-assay responses. The use of assay-specific threshold values in the BDPTs also allows for assay responses to be compared across different trials. Visual comparisons across assays are also facilitated, because all variables share the comparable scaling and the rank order of responses from high to low (i.e. monotonicity) is preserved. However, the biological interpretation of cross-assay comparisons remains a challenge just as with non-transformed data. Another key element in our framework is the hierarchical organization and visual display of immune response data, a strategy that has gained substantial traction for unsupervised learning in high dimensional data analyses [29], to generate “immune response landscapes.”

Our approach provides new insights for each of the five objectives noted above, three of which we highlight briefly. First, comparison of immune response patterns defined by both anti-V1V2 IgG/IgG3 responses and CD4+ T-cell responses between the two vaccine regimens showed that a sizable subgroup of RV144 vaccine recipients had robust and broad V1V2-targeting antibody responses yet very low CD4+ T-cell responses. In contrast, a subgroup of HVTN 100 vaccine recipients had very strong CD4+ T-cell responses yet wide variability across antigens in V1V2-targeted antibody responses. Relatedly, the analysis revealed a cluster of about 40% of HVTN 100 vaccine recipients with broad antibody and CD4+ T-cell co-responses. Thus, the P_C_/MF59 regimen could have an advantage for efficacy in HVTN 702, if such co-responses are important for protection. Second, the vaccine landscape analysis facilitated identification of subgroups with rare multivariate response patterns: e.g. vaccine recipients with a complete absence of a response despite receiving all vaccinations, or on the other extreme vaccine recipients with high response breadth across all immune response variables covering many antigen specificities for both humoral and cellular responses. Such an output would flag unusual participants for an independent adjudication. Third, the analysis of HVTN 100 showed a broad dynamic range of immune responses defined by anti-V1V2 IgG/IgG3 antibodies and/or Env-specific CD4+ T cells, suggesting that there will be high power for studying correlates and co-correlates of risk based on these assays. In contrast, efficacy trials of these regimens will not be able to study immune responses defined by anti-gp120/gp140 antibodies and/or Env-specific CD8+ T cells as correlates or as co-correlates of risk due to low overall dynamic ranges.

## Results

### Study participants

All participants selected for immunogenicity assessment remained HIV-uninfected and received all 4 planned immunizations through Month 6 (defined as per-protocol). Peripheral blood mononuclear cells (PBMCs) and plasma (RV144) or serum (HVTN 097, HVTN 100) samples were collected from each selected participant at 2 weeks after the Month 6 vaccination and were used to assess immune responses for the current analysis. We used data from 24 placebo recipients and 201 vaccine recipients from the RV144 trial, 18 placebo recipients and 73 vaccine recipients from the HVTN 097 trial, and 37 placebo recipients and 185 vaccine recipients from the HVTN 100 trial for this assessment (Table 1). The vaccine groups of each study did not significantly differ by age, BMI or sex-at-birth (all p-values >0.05) (Table 1). While all three trials enrolled both males and females, each vaccine arm had a higher percentage of male than female vaccine recipients − 61%, 55%, and 61%, respectively. In South Africa, women generally have a higher BMI than men [30]. BMI data were not collected in RV144.

**Table 1:** Demographic information by treatment assignment (placebo vs vaccine) for RV144, HVTN 097, and HVTN 100 per-protocol participants.

		RV144		HVTN 097		HVTN 100	
n (%)		Placebo	Vaccine	Placebo	Vaccine	Placebo	Vaccine
		24 (100%)	201 (100%)	18 (100%)	73 (100%)	37 (100%)	185 (100%)
Age (y)	18–20	4 (16.7)	56 (27.9)	7 (38.9)	27 (37.0)	8 (21.6)	44 (23.8)
	21–25	14 (58.3)	97 (48.3)	8 (44.4)	29 (39.7)	18 (48.6)	78 (42.2)
	≥ 26	6 (25.0)	48 (23.9)	3 (16.7)	17 (23.3)	11 (29.7)	63 (34.1)
Sex-at-birth	Female	12 (50.0)	79 (39.3)	9 (50.0)	33 (45.2)	18 (48.6)	73 (39.5)
	Male	12 (50.0)	122 (60.7)	9 (50.0)	40 (54.8)	19 (51.4)	112 (60.5)
Male BMI*	Underweight	nc	nc	1 (11.1)	11 (27.5)	2 (10.5)	15 (13.4)
	Normal	nc	nc	8 (88.9)	23 (57.5)	15 (78.9)	79 (70.5)
	Overweight	nc	nc		5 (12.5)	2 (10.5)	16 (14.3)
	Obese	nc	nc		1 (2.5)		2 (1.8)
Female BMI	Underweight	nc	nc		3 (9.1)		5 (6.8)
	Normal	nc	nc	3 (33.3)	12 (36.4)	4 (22.2)	25 (34.2)
	Overweight	nc	nc	1 (11.1)	12 (36.4)	8 (44.4)	25 (34.2)
	Obese	nc	nc	5 (55.6)	6 (18.2)	6 (33.3)	18 (24.7)
Race/Ethnicity	Thai Asian	24 (100)	201 (100)				
	Black			18 (100)	73 (100)	37 (100)	185 (100)

1) BMI data, body mass index (kg/m^2^), are missing for 4 placebo recipients in HVTN 097 and 1 vaccine recipient in HVTN 100. The “underweight”, “normal”, “overweight”, and “obese” categories correspond to BMI values of <18.5, 18.5–25, 25–30, and ≥30, respectively.

2) nc, not collected. BMI data were not collected in RV144.

### Immune response landscapes

The immune response measurements that were common across the trials included 20 intracellular cytokine staining (ICS) measurements of gp120-specific CD4+ or CD8+ T-cell responses and 44 antibody responses as assessed by the binding antibody multiplex assay (BAMA) (S1 Table). Utilizing globally normalized and transformed immune responses, we performed two-way hierarchical clustering to organize participants and immune response variables based on their similarity. The resulting heatmaps allow for valid quantitative comparisons across participants (rows) with respect to multiple immune responses (columns). Across columns, all ICS and BAMA responses underwent BDPT onto a comparable scale. While negative values from -2 to 0 correspond to non-responders, most of the positive transformed values (corresponding to responders) are less than 10. Values larger than 10 were truncated to 10 when constructing the heatmaps to facilitate visual interpretation. Euclidean distances were calculated (on BDPT-transformed scales) between all pairs of immune measures as well as between all participants, enabling two-way clustering and facilitating visualization of ICS and BAMA responses on the same heatmap. Given the monotonic nature of BDPT (i.e., the quantitative order of transformed values is the same as their original values), transformed values within an assay are directly comparable; comparisons across assays can reveal patterns of within-assay relative highs and lows, but the direct comparison needs to be cautious.

**Fig 1:**
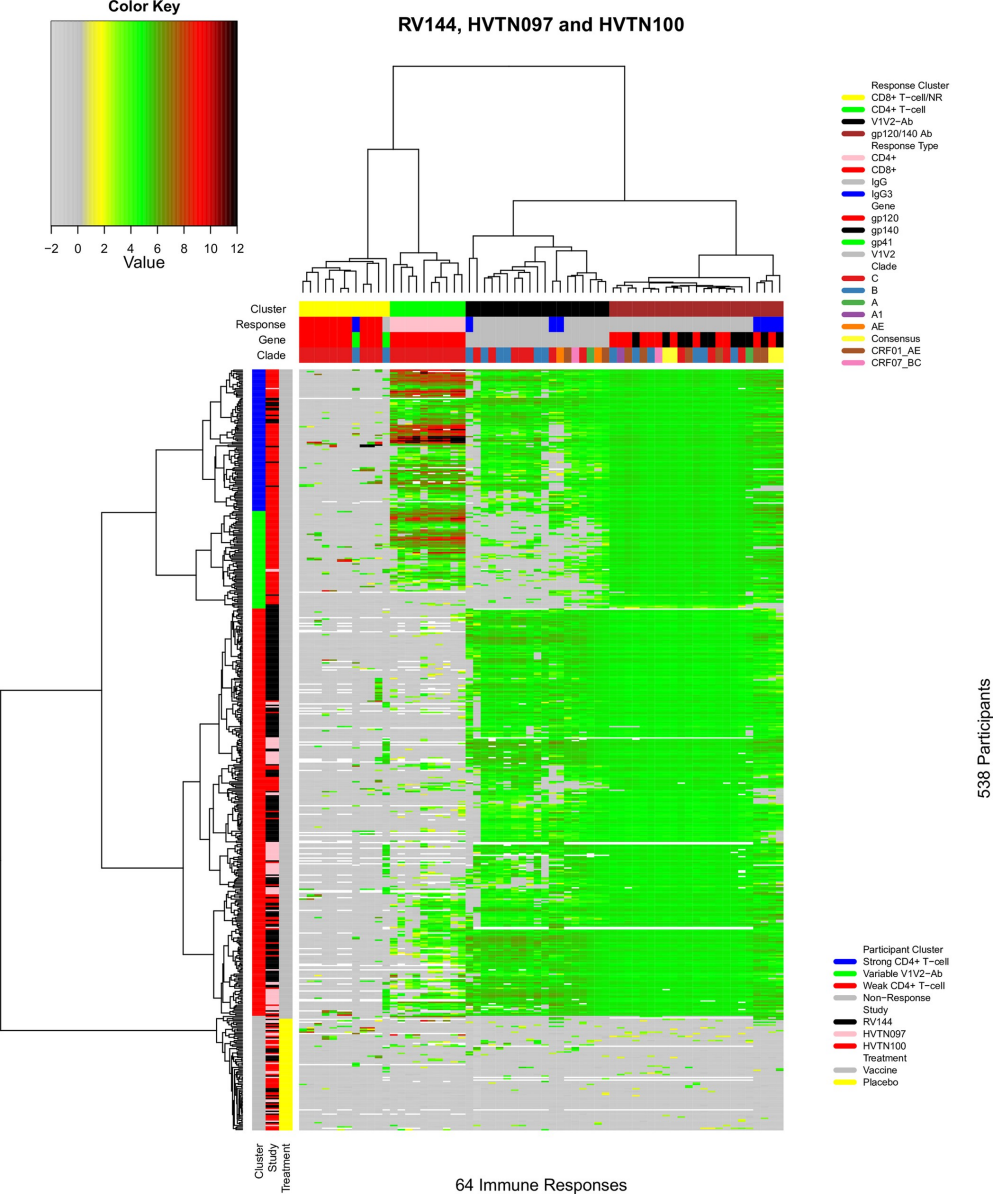
Landscape of immune responses to the P_AE/B_/alum and P_C_/MF59 regimens. The heatmap was generated by two-way hierarchical clustering of participants and immune response variables based on their similarity after bi-directional power transformation of the immune response measurements. The 64 immune response measurements shared across RV144, HVTN 097, and HVTN 100 participants were used. Columns designate immune responses; cluster, response, gene, and clade information are given in the top 4 color-coded bars. Rows designate participants; cluster, study, and treatment assignment information are given in the three color-coded bars on the left. Dendrograms on the top and left illustrate column and participant clustering, respectively. Immune response measurement values are designated by color according to the key shown in the upper left.

Fig 1 shows the landscape of immune responses to P_AE/B_/alum and P_C_/MF59. The 64 immune response variables were grouped into four column clusters. The first cluster of responses included mostly CD8+ T-cell responses, together with one IgG and one IgG3 response, and was characterized by uniformly low values across vaccine recipients; we refer to it as the “CD8+ T-cell/NR” (non-response) cluster. The adjacent cluster included only CD4+ T-cell responses (“CD4+ T-cell” cluster). In this cluster, all CD4+ T-cell response variables showed substantial variation across vaccine recipients and were highly correlated with each other (as demonstrated later). The next column cluster consisted of anti-V1V2 IgG and IgG3 antibody responses (“V1V2-Ab” cluster). Wide variability was observed across V1V2 responses in this cluster. The far-right cluster consisted entirely of anti-gp120 and anti-gp140 IgG and IgG3 responses (“gp120/gp140 Ab” cluster). Notably, all response variables in this cluster were elevated with limited variation among vaccine recipients.

**Fig 2:**
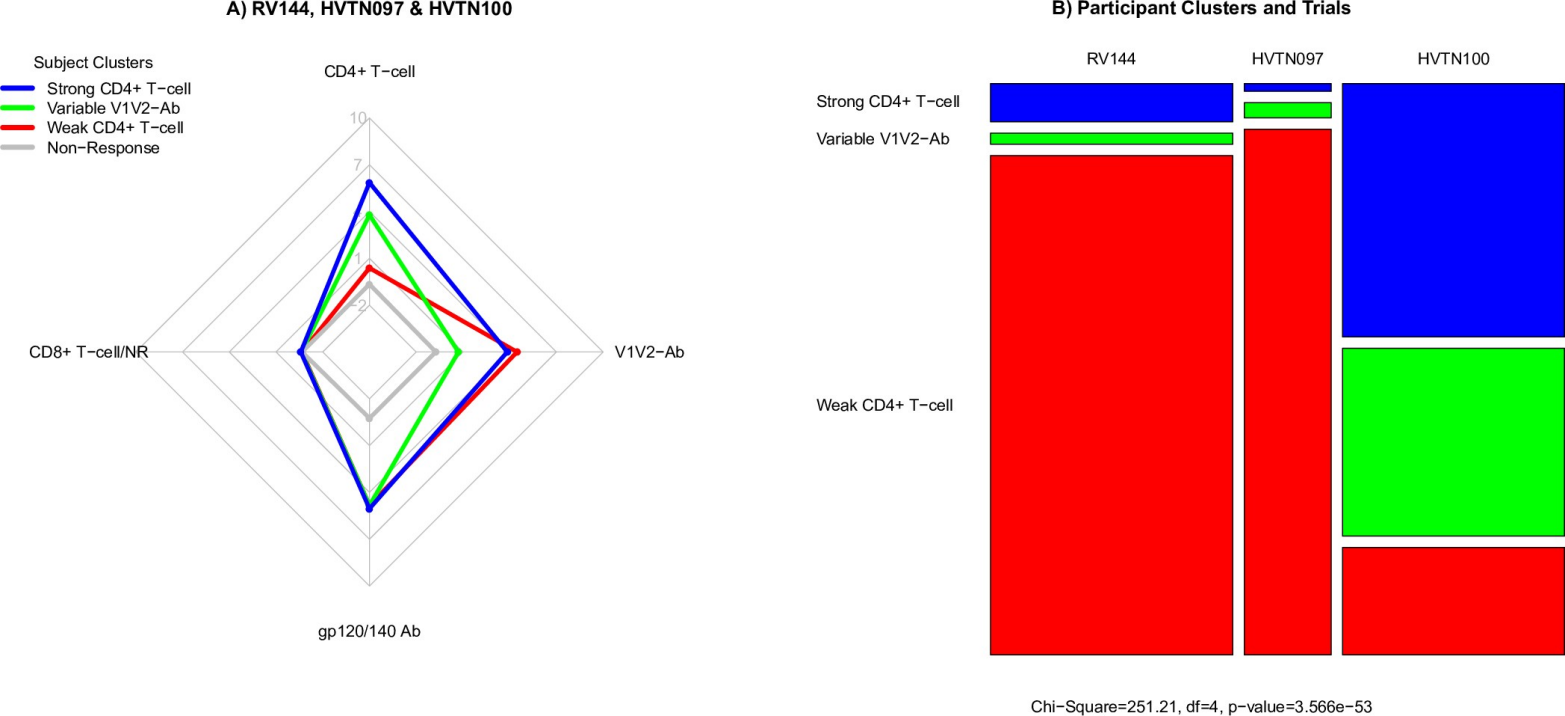
Summary of participant cluster immune response patterns and distribution of participant clusters across studies. A) Radar plot showing the distribution of immune responses in each of the four participant clusters shown in Fig 1. Each colored line represents one participant cluster and each vertex represents an immune response. B) Distribution of participant clusters across the RV144, HVTN 097, and HVTN 100 trials. Each column represents one trial; participant clusters are designated on the left-hand side and are color-coded.

The trial participants were grouped into four distinct clusters. From top to bottom, the first participant cluster included individuals whose vaccine response was characterized by a robust CD4+ T-cell response (“Strong CD4+ T-cell” cluster, blue). Participants in the adjacent cluster had more variable V1V2-Ab responses (“Variable V1V2-Ab” cluster, green), while retaining strong CD4+ T-cell and anti-gp120 responses. The next adjacent cluster, the largest of all four participant clusters, had a relatively weak CD4+ T-cell response (“Weak CD4+ T-cell” cluster, red). The bottom “non-response” cluster included all placebo recipients together with two vaccine recipients. As expected, almost none of the measured immune responses were detected in any of the placebo recipients. The basic immune response patterns of the participant clusters were recapitulated in a radar plot of the data (Fig 2A). All four participant clusters tended to have limited CD8+ T-cell responses. Three of the vaccine recipient clusters showed consistently elevated anti-gp120 and anti-gp140 IgG and IgG3 antibody responses. In contrast, the CD4+ T-cell and V1V2-Ab responses were more varied across the participant clusters.

We next tested whether participant clusters were distributed differently across the studies, after excluding those non-responses, and found highly significant differences (Chi-squared test; P-value < 0.001) (Fig 2B). Visual inspection suggested that these differences were driven mostly by the difference in vaccine regimens (P_AE/B_/alum vs P_C_/MF59); the difference in cluster distribution across the two P_AE/B_/alum study populations (RV144 and HVTN 097) was not significant (P-value = 0.219).

The immune response landscape of the 538 participants across the RV144, HVTN 097, and HVTN 100 trials, using the full set of 307 immune responses, is shown in Panel A of S2 Fig. Missing values (white) are present, because many immune measurements were generated for only one of the trials. Trial-specific immune response landscapes for RV144 (Panel B of S2 Fig), HVTN 097 (Panel C of S2 Fig), and HVTN 100 (Panel D of S2 Fig) were also generated.

### Comparison of the P_AE/B_/alum regimen in Thailand and South Africa

**Fig 3:**
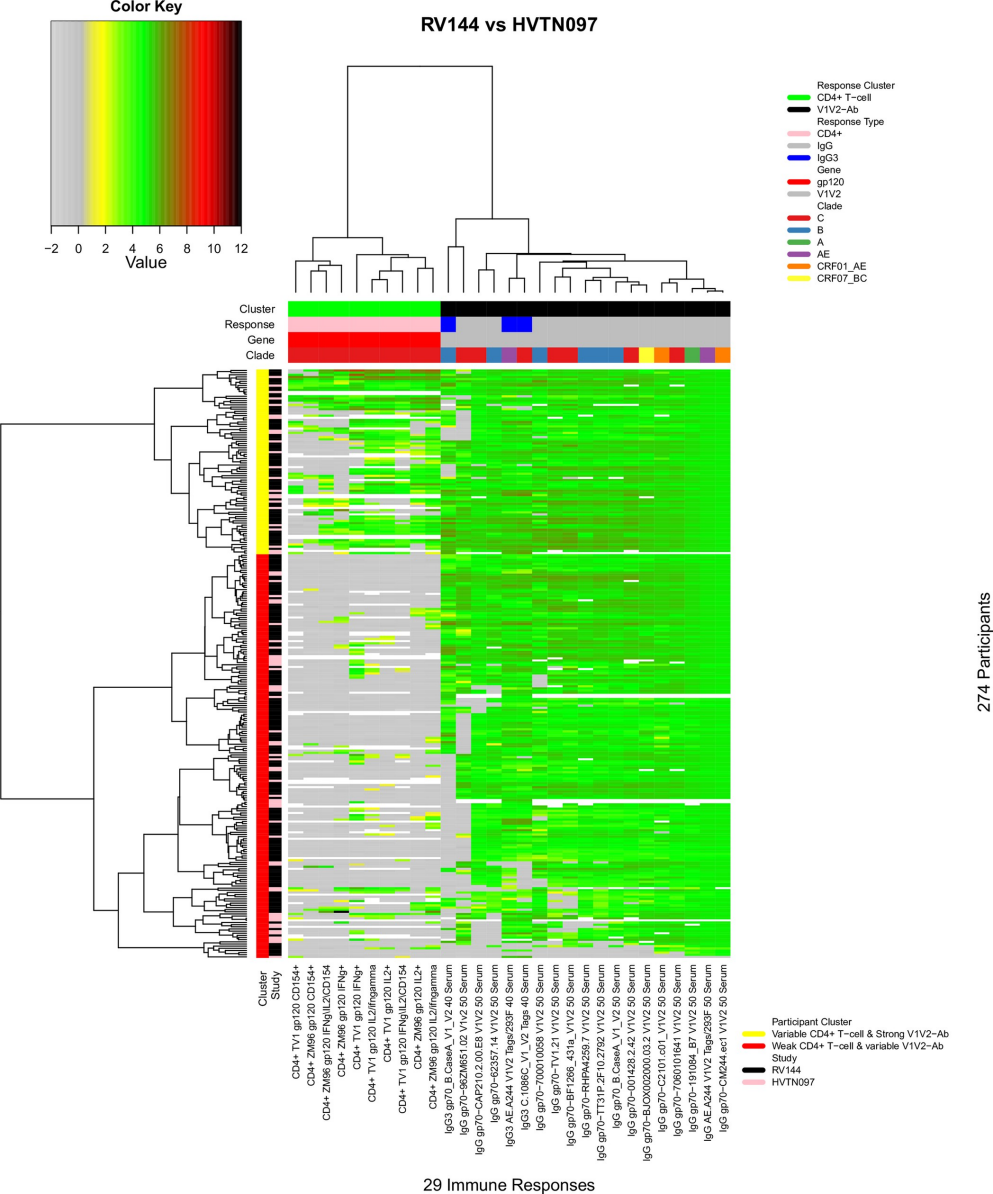
Landscape of immune responses in Thai and South African populations to the P_AE/B_/alum regimen. The heatmap shows 29 selected immune response measurements of RV144 and HVTN 097 vaccine recipients. Columns designate immune responses; cluster, response, gene, and clade information are given in the top 4 color-coded bars. Rows designate participants; cluster and study information are given in the two color-coded bars on the left. Dendrograms on the top and left illustrate column and participant clustering, respectively. Immune response measurement values are designated by color according to the key shown in the upper left.

Comparison of the immune response landscapes between RV144 and HVTN 097 enabled conclusions to be drawn about the responses elicited by the P_AE/B_/alum regimen in a Thai population versus the same regimen in a black South African population. We compared these two trials excluding some of the immune response variables included in Fig 1, namely CD8+ response variables because of generally absent/low responses, and gp120/140-Ab response variables due to their consistently elevated levels and low variability. Excluding these response clusters netted 29 response variables in the CD4+ T-cell and V1V2-Ab clusters for further analysis. We also excluded placebo recipients, all of whom were in the non-response cluster. The participants and responses were then re-clustered (Fig 3). As expected, the immune responses were grouped into CD4+ T-cell and V1V2-Ab clusters, and the participants were grouped into two row clusters: the top participant cluster included participants who tended to have variable CD4+ T-cell responses and strong anti-V1V2 IgG and IgG3 antibody responses (“Variable CD4+ T-cell & strong V1V2-Ab” cluster). Meanwhile, participants in the bottom cluster tended to have weak CD4+ T-cell responses and variable anti-V1V2 IgG and IgG3 antibody responses (“Weak CD4+ T-cell & Variable V1V2-Ab” cluster). Both RV144 and HVTN 097 vaccine recipients appeared to cluster randomly into these two participant clusters (Chi-squared test; P-value = 1.00).

### Comparison of the P_AE/B_/alum and P_C_/MF59 regimens

**Fig 4:**
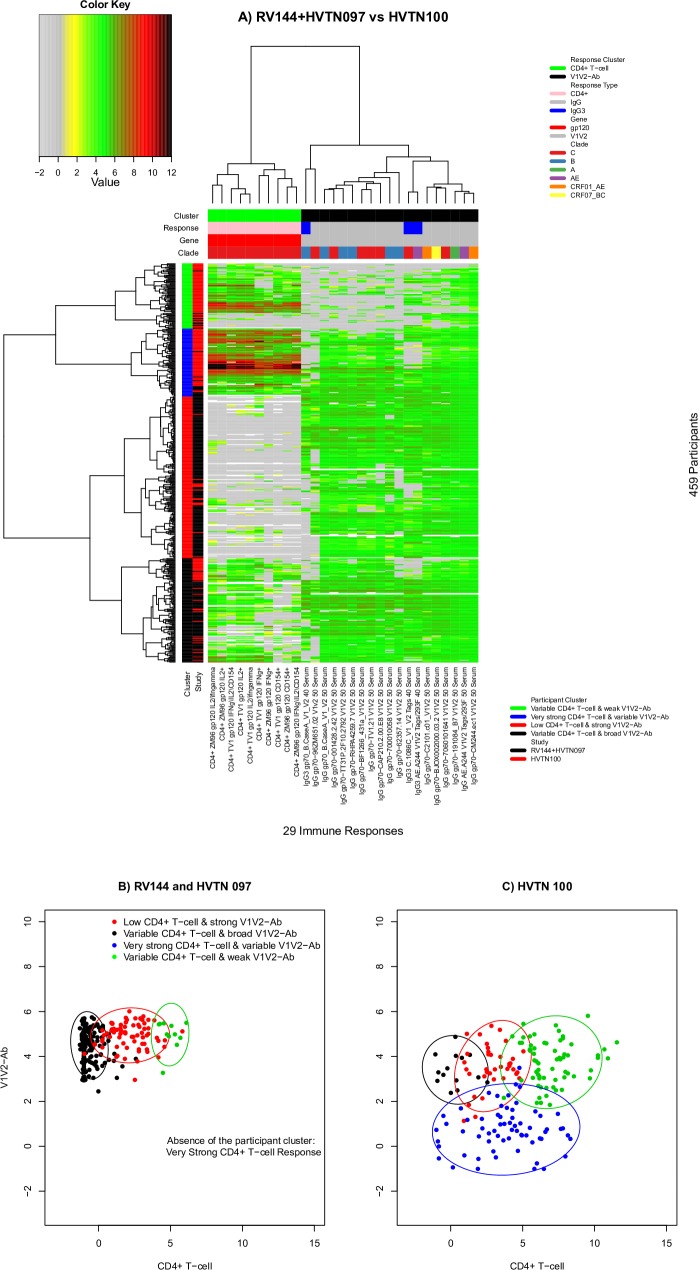
Immune response landscapes for the P_AE/B_/alum and P_C_/MF59 regimens. A) Heatmap of the same 29 immune response measurements combining RV144 and HVTN 097 vaccine recipients (pooled) and HVTN 100 vaccine recipients. Columns designate immune responses; cluster, response, gene, and clade information are given in the top 4 color-coded bars. Rows designate participants; cluster and study information are given in the two color-coded bars on the left. Dendrograms on the top and left illustrate column and participant clustering, respectively. Immune response measurement values are designated by color according to the key shown in the upper left. Scatterplots of participant-specific average values of CD4+ T-cell and anti-V1V2 antibody responses in (B) RV144 and HVTN 097 and (C) HVTN 100. Ellipsoids of bivariate normal distributions, based on respective empirical means and co-variances, are used to approximately cover 90% empirical bivariate observations. Panels B and C are shown on BDPT-transformed scales.

Given the lack of evidence of a significant difference between the RV144 and HVTN 097 immune response landscapes, we pooled data from these two trials and compared them with data from the HVTN 100 trial, to assess whether there were landscape differences between the P_AE/B_/alum and P_C_/MF59 vaccine regimens. We used the same set of participants and the same 29 immune responses that showed substantial variation among vaccine recipients in all three trials and performed unsupervised learning and clustering (Fig 4A). Again, the CD4+ T-cell and V1V2-Ab response clusters were distinct. The 459 vaccine recipients were grouped into four participant clusters: the top cluster (green) included participants who had relatively weak anti-V1V2 IgG and IgG3 antibody responses (“Variable CD4+ T-cell & weak V1V2-Ab”). The adjacent cluster (blue) included a group of participants whose CD4+ responses tended to be fairly strong with a variable anti-V1V2 IgG and IgG3 response (“Very strong CD4+ T-cell & variable V1V2-Ab"). The next adjacent participant cluster (red) included participants who had relatively low CD4+ T-cell response to absent and whose anti-V1V2 IgG and IgG3 responses were relatively strong (“Low CD4+ T-cell & strong V1V2-Ab”). The bottom participant cluster had variable CD4+ T-cell responses and broadly elevated anti-V1V2 IgG and IgG3 responses (“Variable CD4+ T-cell & broad V1V2-Ab”).

Bekker et al. [26] noted that HVTN 100 participants had lower binding antibody responses to many V1V2 antigens compared to RV144. The landscape analysis provided additional insights about the CD4+ T-cell and V1V2-specific Ab co-response. For example, three additional observations can be made from Fig 4A: (a) almost all vaccine recipients with both strong CD4+ T-cell and strong V1V2-Ab responses received P_C_/MF59 (blue, participant cluster “Very Strong CD4+ T-cell & variable V1v2-Ab”); (b) almost all recipients of either vaccine who lacked CD4+ T-cell responses had medium V1V2-Ab responses (red, participant cluster "Low CD4+ T-cell & strong V1V2-Ab"), and, for P_AE/B_/alum recipients in this cluster, many had breadth of antibody responses to almost all of the V1V2 antigens; and (c) only P_C_/MF59 recipients sometimes lacked V1V2-Ab responses yet had medium-to-strong CD4+ T-cell responses (green, participant cluster "Variable CD4+ T-cell & weak V1V2-Ab").

The above analysis revealed that the bottom two vaccine recipient clusters appeared to be enriched for RV144 and HVTN 097 vaccine recipients, whereas the top two clusters appeared to be enriched for HVTN 100 vaccine recipients. Consistent with this observation, vaccine recipient cluster membership was significantly associated with trial membership in RV144 or HVTN 097 versus HVTN 100 (Chi-squared test P-value = 2.2x10^-16^). This significant association indicated that P_C_/MF59 tended to elicit higher CD4+ T-cell responses, yet lower V1V2-Ab responses than P_AE/B_/alum.

To gain further insight into the bivariate distribution of CD4+ T-cell and V1V2 Ab response we generated two-dimensional scatter plots of the participants in RV144 plus HVTN 097 (Fig 4B) and HVTN 100 (Fig 4C), colored by participant clusters. For each participant, an average CD4+ T-cell (x-axis) response value and an average V1V2 Ab response value (y-axis) were computed (Fig 4B and 4C). They revealed that while the distributions of the V1V2-Ab response values were similar across response clusters in RV144+HVTN 097 versus HVTN 100, the CD4+ T-cell responses generally showed a greater range in HVTN 100, particularly for the “Variable CD4+ T-cell & weak V1V2-Ab” cluster, where the CD4+ T-cell responses ranged much higher in magnitude in HVTN 100.

### Vaccine recipients with unique immune response profiles

The clustered multivariate immune response landscapes enabled identification of vaccine recipients with unique immune response patterns, such as per-protocol vaccine recipients with complete “non-take” (no or limited immune responses across all variables, thus clustering with placebo recipients) despite having received all 4 immunizations through Month 6. We observed two such vaccine recipients: one who participated in HVTN 097 and the other who participated in HVTN 100 (Fig 1). Both were male and black, with BMI values of 17.5 and 17.4, putting each at the 11% and 5% percentile of all males in the respective trials.

**Fig 5:**
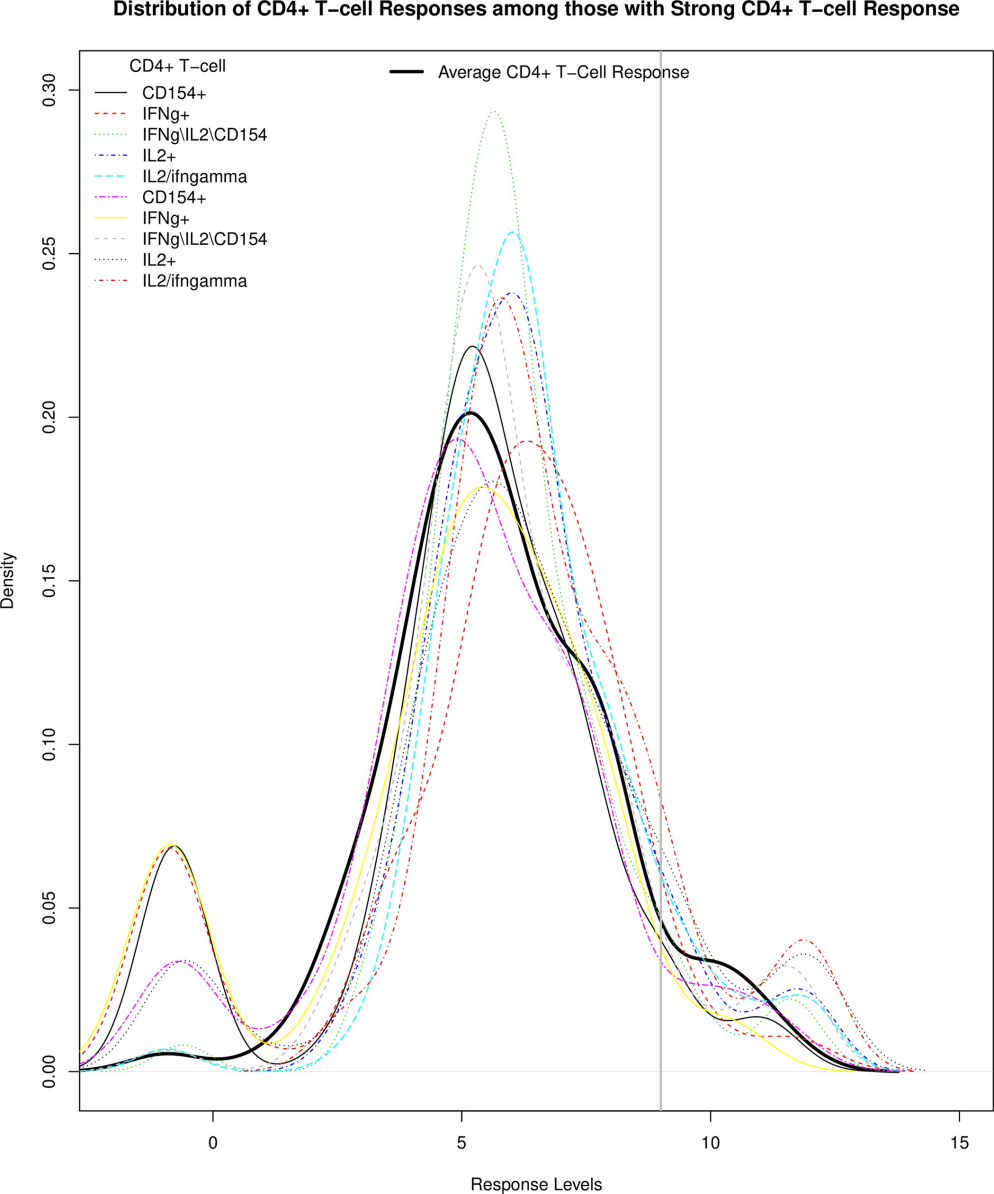
Distribution of CD4+ T−cell response magnitude (BDPT-scaled) among 100 participants in all three trials with strong CD4+ T−cell responses. The densities of the different CD4+ T-cell responses (color coded) and the average CD4+ T-cell response are shown by response level.

Of equal interest are vaccine recipients with an unusually strong and consistent immune response pattern. For example, 97 P_C_/MF59 recipients (the top participant cluster in Fig 1) were clustered together with the “Strong CD4+ T-cell” cluster, and had strong CD4+ T-cell responses, in addition to gp120-Ab and V1V2-Ab responses. We noted that the average CD4+ T-cell response probability density appeared to center on ~5, and 8 vaccine recipients fell in the right tail with the threshold value of 9 (Fig 5). Density distributions of these 8 individual CD4+ T-cell responses showed the “Strong CD4+ T-cell” with their response levels approaching 15. Interestingly, all vaccine recipients were male and all of them participated in HVTN 100. They also tended to have a normal BMI (7 normal and 1 overweight), although their distribution did not differ significantly from that of the others (p = 0.22).

### Associations of demographics with cluster membership

Based on the CD4+ T-cell, gp120-Ab and V1V2-Ab response clusters, all participants were clustered into four participant groups: Strong CD4+ T−cell, Variable V1V2−Ab, Weak CD4+ T−cell and non-response. Age, sex-at-birth, and BMI were tested for an association with the immunologically-defined clusters. None of the three covariates were associated with participant cluster membership (p-value>0.05) (Table 2).

**Table 2:** Results from analyzing cluster membership with three demographic covariates (age, sex-at-birth and BMI), from 538 vaccine and placebo recipients in all three trials with 64 immune responses (shown in Fig 1).

Participant Cluster Membership		Strong CD4+ T-cell	Variable V1V2-Ab	Weak CD4+ T-cell	Non-Response	P-value^1^
	n (%)	100 (100%)	69 (100%)	288 (100%)	81 (100%)	
**Age (y)**	18–20	23 (23)	17 (24.6)	87 (30.2)	19 (23.5)	0.223
	21–25	51 (51)	28 (40.6)	123 (42.7)	42 (51.9)	
	≥26	26 (26)	24 (34.8)	78 (27.1)	20 (24.7)	
**Sex-at-birth**	Female	37 (37)	31 (44.9)	117 (40.6)	39 (48.1)	0.223
	Male	63 (63)	38 (55.1)	171 (59.4)	42 (51.9)	
**BMI (kg/m**^**2**^**)**	(0,18.5]	9 (10.5)	5 (7.7)	18 (17.1)	5 (8.8)	0.222
	(18.5,25]	46 (53.5)	33 (50.8)	60 (57.1)	30 (52.6)	
	(25,30.5]	21 (24.4)	18 (27.7)	19 (18.1)	11 (19.3)	
** **	(30.5,100]	10 (11.6)	9 (13.8)	8 (7.6)	11 (19.3)	

^1^Each p-value was computed by 100,000 permutations.

### Assays and antigens with uniform lack of vaccine response

The immune response landscapes also facilitated identification of immune response variables that were absent across all (or almost all) vaccine recipients. For example, the CD8+ T-cell column cluster (Fig 1) included 12 such immune responses, including ten gp120-specific CD8+ T-cell variables and two gp41-specific antibody responses (IgG and IgG3) (S2 Table). Prior to normalization and transformation, the original CD8+ T-cell responses indicated that across participants the median percent of CD8+ T-cells expressing cytokines was 0% and the mean and median levels of gp41-specific IgG and IgG3 were 5.5 and 3.3, respectively; after normalization and transformation, the median values of all 12 responses were near −1.00.

### Dynamic ranges of immune responses

**Fig 6:**
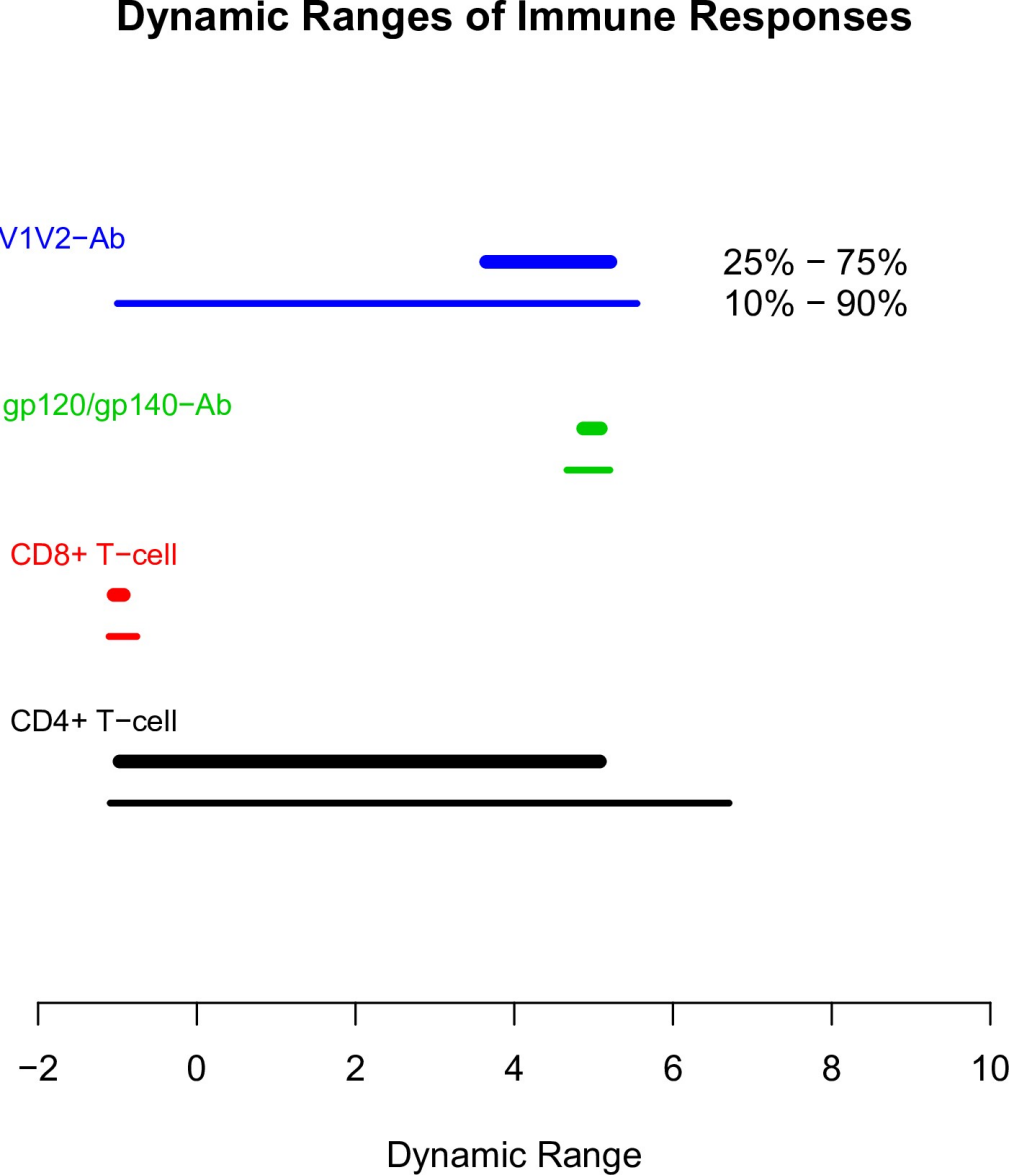
Dynamic ranges of the 64 immune responses shared across the three trials. The ranges of the immune responses after bi-directional power transformation are shown (25^th^ percentile to 75^th^ percentile, thicker lines; 10^th^ percentile to 90^th^ percentile, thinner lines). Lines are color-coded by response type.

Among BAMA readouts, the interquartiles of anti-V1V2 IgG/IgG3 antibody responses were modest, ranging from 3.6 to 5.2, while their 10% and 90% quantiles were much wider, from -1 to 5.5 (Fig 6). On the other hand, BAMA readouts from anti-gp120 IgG/IgG3 antibody responses had narrow dynamic ranges, from 4.6 to 5.2 with the 10% and 90% quantiles, respectively. For ICS readouts, CD4+ T-cell responses had large dynamic ranges with the interquartile (-0.97, 5.1) and 10%-90% quantiles (-1.1, 6.7). In contrast, CD8+ T-cell responses had 10% and 90% quantiles of (-1.1, -0.8).

### Redundant immune responses

**Fig 7:**
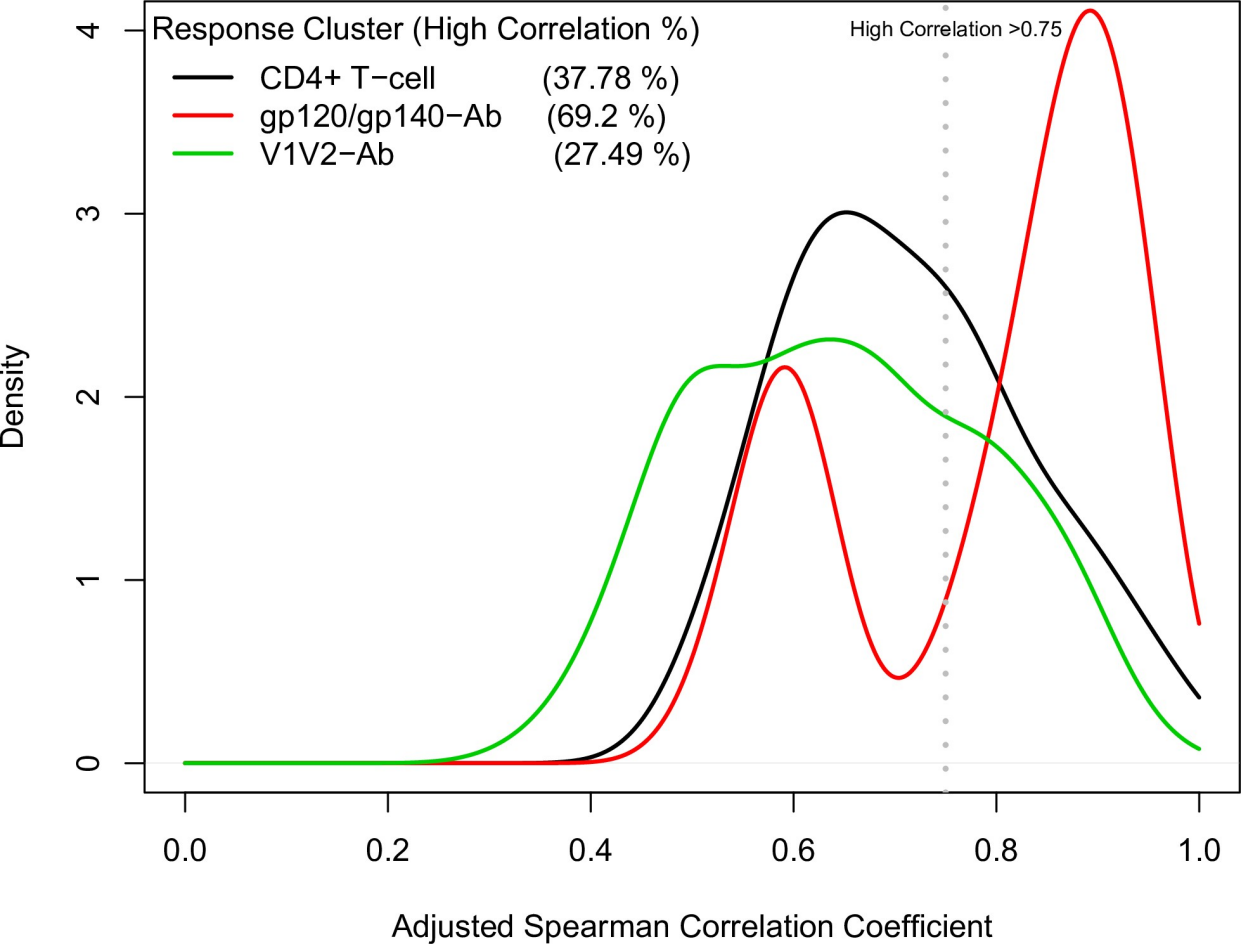
Distributions of study-adjusted rank-based Spearman correlations between immune variables within each of the three immune response clusters. Correlations were computed across participants in all three studies. The density (y-axis) indicates the relative number of immune response variable pairs with a given correlation (x-axis). “High correlation” was defined as a correlation greater than the threshold value of 0.75 (dashed line).

Applying a rank-based method, we computed pairwise correlation coefficients between all immune responses within each immune response cluster, excluding the CD8+ T-cell response cluster (Fig 7). Correlations were computed across participants in all three studies. The distributions of the estimated Spearman correlations for responses in the CD4+ T-cell cluster and those in the V1V2-Ab cluster each consisted of a wide single peak, centered around 0.6~0.7. Using a threshold for high correlation of 0.75, 38% and 27% pairs of the immune responses in these two clusters were highly correlated. Interestingly, the correlation distribution of responses in the gp120/gp140-Ab cluster appeared to be bimodal, with one peak centering on 0.6 and a second peak around 0.9, with the latter peak including highly correlated gp120/gp140-Ab responses. Using the same high correlation threshold of 0.75, 69% of the pairs of immune response measurements in this cluster were highly correlated.

### Analysis of vaccine-matched immune responses

**Fig 8:**
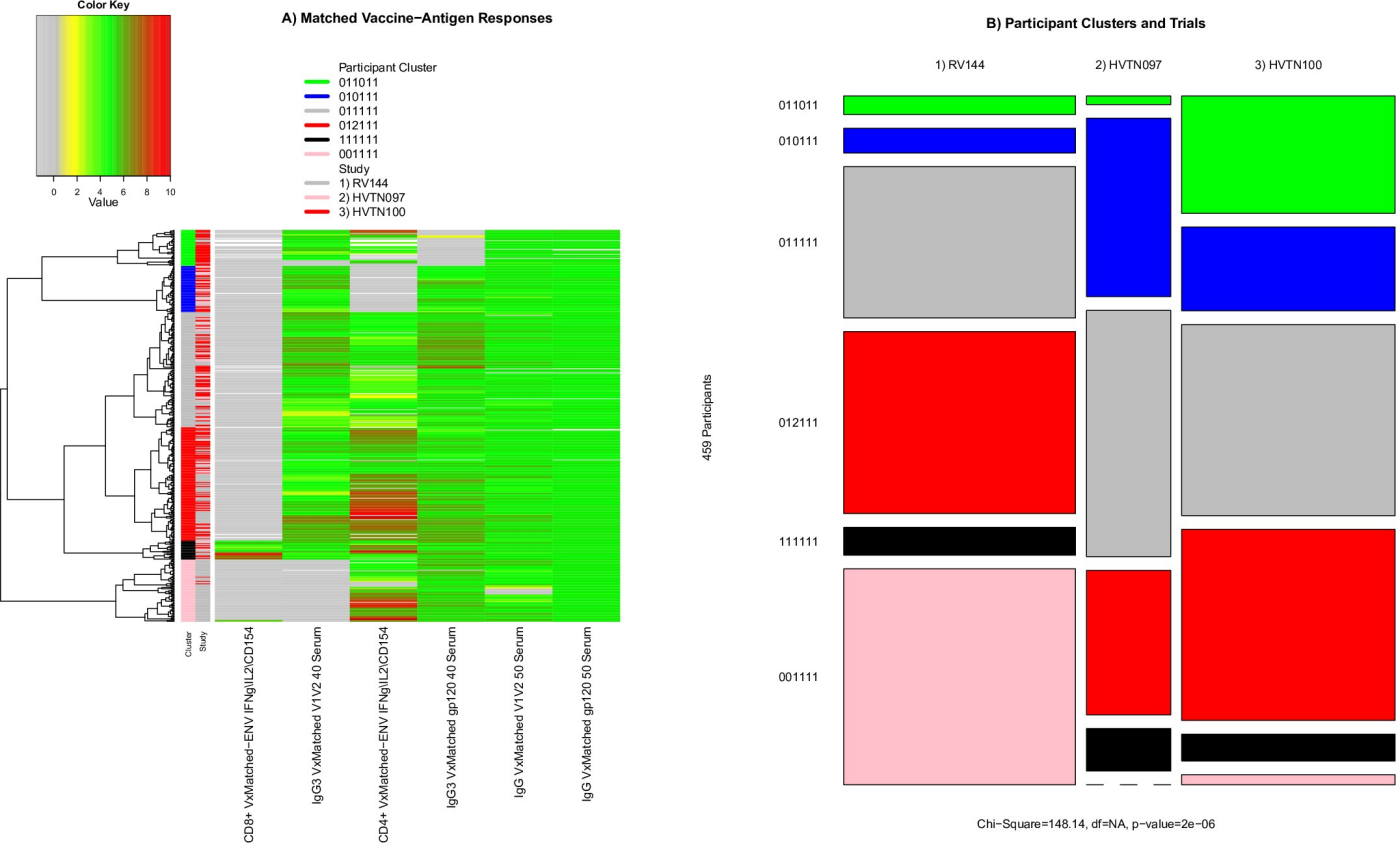
Immune response landscape of vaccine-matched responses and participant clustering based on vaccine-matched immune responses. A) Heatmap showing the vaccine-matched CD8+ T-cell polyfunctionality, anti-V1V2 IgG3 antibody binding, CD4+ T-cell polyfunctionality, anti-gp120 IgG3 antibody binding, anti-V1V2 IgG antibody binding, and anti-gp120 IgG antibody binding measurements used to organize participants into the six row clusters. B) The distribution of participants in each trial (column) in each cluster is represented by the row height. Box size is proportional to the number of participants.

Both ICS and BAMA responses are measured using HIV antigens that may or may not be matched to one of the immunogens in each of the vaccine regimens. We identified six classes of BAMA or ICS measurements that were made using at least one vaccine-matched antigen for each of the three trials: (1) Env-specific IFN-γ\IL-2\CD154+ CD4+ T-cell, (2) Env-specific IFN-γ\IL-2\CD154+ CD8+ T-cell, (3) anti-gp120 IgG binding, (4) anti-gp120 IgG3 binding, (5) anti-V1V2 IgG binding, and (6) anti-V1V2 IgG3 binding. Based on these vaccine-matched immune responses, participants were hierarchically organized into six row clusters (Fig 8A). Similar to the analyses above, we tested whether participants in the three trials were distributed proportionately among the six participant clusters (Fig 8B). We assigned each cluster a six-digit number in which each position takes the value of 0, 1 or 2, corresponding to a low, high or highest response, for each of the six vaccine-matched immune responses. For instance, the top participant cluster, 011011, consisted of participants with low CD8+ T-cell, high IgG3-V1V2, high CD4+ T-cell, low IgG3-gp120, high IgG-V1V2, and high IgG-gp120 responses. The second cluster from the top shared the same response pattern as the top cluster, with the exception of low CD4+ T-cell and high IgG3-gp120 responses. The next two clusters, 011111 and 012111, all had high responses except for CD8+ T-cell, with the 012111 cluster having a much higher CD4+ T-cell response than the 011111 cluster. Cluster 111111 had high responses for all six vaccine-matched responses. Cluster 001111 had low CD8+ T-cell and IgG3-V1V2 responses, while all other responses were elevated. Across these six participant clusters, cluster membership was significantly different across three trials (Chi-squared p <0.001). Moreover, pairwise comparisons found significant differences among all trials (all p <0.001). These differences seemed to be driven by distinct clusters of non-responders for the IgG3 antibody and CD4+ T-cell responses. For example, one cluster of HVTN 100 vaccine recipients had very low gp120-specific IgG3 responses and one cluster of RV144 vaccine recipients had very low V1V2-specific IgG3 responses. In contrast, these low responses were not generally associated with low gp120 or V1V2-specific IgG responses, which were much less variable across the clusters. The CD4+ T-cell polyfunctionality score also contributed to differences between the trials. A cluster of mostly HVTN 097 participants had very low CD4+ T-cell responses while clusters of HVTN 100 participants had the highest responses; these observations were similar to those based on combined analyses of vaccine-matched and mismatched responses.

## Discussion

Our multivariate unsupervised learning and visualization approach differs from univariate approaches that have previously been applied to data from these trials in several ways. Instead of examining how different vaccine regimens may elicit shifts in population-level parameters of individual measures, i.e. assessing changes in response mean or median values, our approach considered the entire multivariate immune response profile. This approach enabled us to evaluate if subgroups of participants sharing similar vaccine-induced immune response profiles could associate with key meta-groups of interest (defined by vaccine regimen or demographics) and that the frequencies of these subgroups differed across meta-groups. Identification of participant subgroups was enabled by integration of diverse immune response measures; similarly, combining data from multiple trials revealed how immune response measures were related to one another. Using our approach, the participant clusters and the immune response measurements defining each cluster generate hypotheses that can be tested in future experimental and computational analyses, with the aim of better understanding the mechanisms underlying response heterogeneity. As vaccine efficacy is a complex set of interrelated immune responses, our approach puts individually evaluated immune responses into an interrelated and multivariate framework allowing one to interrogate these relationships more comprehensively.

By comparing the immune response landscapes of the P_AE/B_/alum regimen in the RV144 versus HVTN 097 trials, we first recapitulated the findings of Gray et al. [25] − that the landscapes are comparable between the Thai and South African populations, with most vaccine recipients exhibiting broad V1V2-specific antibody responses. In addition, through multivariate analysis of antibody and T-cell responses, we found substantial variability in the joint responses, including a small subgroup with complete non-take for both antibody and T-cell responses despite receiving all vaccinations, a small subgroup with strong responses across all variables, a large subgroup with a broad V1V2 antibody response combined with no CD4+ T-cell response, and a large subgroup with a broad V1V2 antibody response combined with a strong CD4+ T-cell response.

We also confirmed the individual assay findings of Bekker et al. that V1V2 antibody responses tended to be stronger and broader for the P_AE/B_/alum vaccine regimen compared to P_C_/MF59, whereas the latter regimen elicited stronger and broader CD4+ T-cell responses [26]. Our joint antibody and CD4+ T-cell analysis revealed that almost all vaccine recipients with both strong V1V2 antibody and gp120-specific CD4+ T-cell responses received P_C_/MF59_,_ almost all recipients of either vaccine with absent CD4+ T-cell responses had medium and broad V1V2 antibody responses, and only P_C_/MF59 recipients sometimes lacked V1V2 responses yet showed medium-to-strong CD4+ T-cell responses. Cumulatively, these results demonstrate how the approach enables the characterization and comparison of immunogenicity across vaccine regimens and other participant factors at a greater multivariate resolution than previous analyses.

The work also demonstrates the utility of multivariate unsupervised learning and visualization for the other vaccine development objectives outlined in the Introduction. For instance, we identified two vaccine recipients (one in HVTN 097 and one in HVTN 100) who lacked responses for all assayed immune responses and hence appeared to have “complete non-take” despite having received all vaccinations. In addition to being of biological interest, this finding has value for data control, prompting extra checking of correct administration of treatment assignment. We also identified three separate subgroups of vaccine recipients for whom vaccination induced only V1V2-targeting IgG or IgG3 responses, only CD4+ T-cell responses, or both response sets.

Our investigation of dynamic response ranges of the four response clusters identified in the landscape containing all 64 immune responses shared across the three trials (Fig 1) showed that both gp120-specific CD4+ T-cell responses and V1V2-specific IgG/IgG3 antibody response clusters had broad dynamic ranges that will provide ample statistical power for immune correlates analyses of HVTN 702. In contrast, CD8+ T-cell responses and gp120-specific IgG/IgG3 antibody responses had limited dynamic ranges (uniformly low levels and uniformly high levels, respectively), precluding immune correlates analyses using these responses. Note that while BDPT transformed values were used for the computation, patterns of dynamic ranges for untransformed values were similar (not shown).

Our analysis of vaccine-matched immune responses showed that some of the inter-trial variability we observed based on all responses, could be explained by the comparison of responses to vaccine-matched and -mismatched antigens. For example, the cluster of mostly HVTN 100 participants with relatively weak V1V2-specific IgG and IgG3 Ab responses (Fig 3, “Variable CD4+ T-cell & weak V1V2-Ab” cluster) was not evident in the responses to vaccine-matched V1V2- or gp120-specific IgG. Instead, we observed a clear cluster of RV144 participants who had a very low V1V2-specific IgG3 response. Levels of V1V2-specific IgG3 (both vaccine matched and a clade C antigen) were previously shown to be lower among HIV-1 infected participants in a follow-up case-control study of RV144 [17]. Though the IgG3 responses reported here do not include HIV-1 infected cases from RV144 by design, we hypothesize that the vaccine recipients with very low V1V2-specific IgG3 were at higher risk of infection and that the comparatively low frequency of these participants in HVTN 097 and HVTN 100 may indicate comparatively lower risk of infection. We also found with the vaccine-matched analysis that a much lower proportion of RV144 and HVTN 097 participants lacked a CD4+ T cell response than was apparent in the analysis of all the antigen-specific responses. Though it remained apparent that clusters of HVTN 100 recipients had a greater CD4+ T-cell response compared to RV144 and HVTN 097 participants, the cluster of RV144 and HVTN 097 recipients with no CD4+ T cell response was limited and mostly among RV144 participants. These findings showed that after controlling for antigenic-mismatch, differences between the responses induced in each trial could be identified more precisely and that the differences could be more confidently attributed to differences between the trial populations and vaccine regimens, as opposed to the antigen variants used in the immune assays.

Though a vaccine-matched response is highly relevant for understanding vaccine immunogenicity in different populations or with different adjuvants, it may be equally, if not more, important to understand vaccine-elicited responses to circulating viruses, to which participants in efficacy trials may be exposed. Standardized reagent panels of practical numbers of antigens (9 to 12) have been selected to optimally represent antigenic variability of potential globally circulating viruses [31, 32]; the methods studied here would apply well to data sets emanating from these breadth panels. As multivalent vaccine regimens are developed with the specific goal of eliciting responses to a more diverse breadth of HIV-1 viruses, it will become increasingly important to consider how responses are evaluated and compared.

We also identified clusters of highly correlated (and, therefore, redundant) immune response readouts by studying adjusted correlations of pairs of immune response variables within clusters identified by the landscape. For example, 38% of the pairwise correlation coefficients within the CD4+ T-cell response clusters exceeded 0.75 and thus were highly correlated, and 27% of responses within the V1V2-specific IgG and IgG3 response clusters were highly correlated. These results suggest that many immune response clusters are homogeneous enough that they could be assessed as single variables in immune correlates of risk analyses; this could be an effective way to reduce dimensionality of high-dimensional data and thus minimize a multiplicity testing correction penalty. Further, the correlation pattern may provide a rational way to design future immune response assays.

A limitation of our analysis is that only variables from the BAMA and ICS assays were used. Our analysis was restricted to these assays since both have been standardized and validated and are conducted at centralized labs, giving us high confidence that it is appropriate to directly compare a measurement made on a sample from a participant in one study to the same measurement made on a sample from a different participant in a different study. Also, a large number of individual immune response variables were measured with these assays across RV144, HVTN 097, and HVTN 100. The immune response landscape framework is anticipated to provide more insights as increasing numbers of functional assays are standardized, validated and applied. For example, in the HVTN 505 trial of a DNA recombinant adenovirus 5 HIV vaccine regimen, multiple functional assays have been conducted on vaccine recipient samples including an antibody neutralization assay, a cellular viral inhibition assay, antibody dependent cellular cytotoxicity assays, an antibody dependent cellular phagocytosis assay, an assay determining the breadth of the T-cell response, an antibody avidity assay, and an IgG subclass assay.

While understanding the association of immune response clusters with vaccine protection is the ultimate goal, in this study only RV144 had a sufficiently large sample size and a sufficiently long follow-up duration to be able to assess the vaccine effect on incident HIV-1 infection. Numerous studies (see the Introduction) have reported on the immune responses of RV144 vaccine recipients that were correlates of risk, which included analyses of the data that we analyzed in this study. As we believe that additional post-hoc, re-analysis of these immune measures with respect to HIV-1 infection is unlikely to provide further interpretable benefit to the field, we decided to focus on identifying understanding response heterogeneity, identifying novel biomarker clusters and evaluating their potential in the anticipated correlates of risk analyses of ongoing efficacy studies.

In general, a main application of the framework is the comparison of immune response landscapes among vaccine regimens, with one contemporary future application of interest being the comparison of the two regimens currently in efficacy testing (P_C_/MF59 in HVTN 702 vs. Ad26 Mosaic/gp140/Alum clade C in HVTN 705/VAC89220HPX2008), which would help prepare for the interpretation of the trial results and for the immune correlates of risk/protection analyses. Comparing P_C_/MF59 and Ad26/gp140 vs. DNA/protein vaccine regimens is another contemporary application of interest. In addition, the framework would apply for comparing vaccine regimens/populations by immune response landscapes measured at other time points besides approximate peak after last vaccination, including “durability time points” 6 months or more post last vaccination and “innate time points” with a typically intense phlebotomy scheduling after vaccination. Moreover, the framework would apply for deeper multivariate comparisons that add immune response data from additional compartments beyond blood such as mucosal tissues.

## Methods

### Study populations

We assessed the immune response profiles of HIV vaccine regimens studied in the RV144, HVTN 097, and HVTN 100 trials. Participants who deviated from the protocol, i.e. who did not receive all of the first four scheduled vaccinations or did not have immune response measurements, were excluded.

The RV144 trial was a phase 3 efficacy trial conducted in Thailand that randomized 16,402 participants (1:1 ratio) to ALVAC-HIV prime plus AIDSVAX gp120_B/E_ boost or placebo. Participants in the vaccine arm received ALVAC-HIV at Month 0 and Month 1, followed by ALVAC-HIV+AIDSVAX gp120_B/E_ at Month 3 and Month 6. From this cohort, 212 vaccine recipients and 24 placebo recipients were randomly selected from respective vaccine and placebo arms, and their immune responses were measured at Month 6.5 (2 weeks post last vaccination). Eleven vaccine recipients did not receive all 4 vaccinations and their immune response measurements were excluded from analysis; thus immune responses from 201 vaccine recipients were analyzed here. All placebo recipients received four placebo treatments.

HVTN 097 was a randomized, double-blind, placebo-controlled phase 1b trial of the P_AE/B_/alum vaccine regimen and was conducted in South Africa [25]. A total of 100 participants were randomized (3:1:1 ratio) to receive tetanus+hepatitis B vaccines plus ALVAC-HIV and AIDSVAX gp120_B/E_ prime/boost (T1, n = 60), ALVAC-HIV and AIDSVAX gp120_B/E_ prime/boost (T2, n = 20), or tetanus+hepatitis B vaccines plus placebo (T3, n = 20). Participants in T1 and T2 received ALVAC-HIV at Month 0 and Month 1, followed by ALVAC-HIV+AIDSVAX gp120_B/E_ at Month 3 and Month 6. Participants in T1 and T3 received a tetanus vaccination at Month -1, followed by the hepatitis B series at Months 6.5, 7.5, and 12. Serum samples and PBMCs were collected at Month 6.5 (2 weeks post last HIV-vaccination) for immunogenicity measurements. There were no meaningful differences in any HIV immune responses between participants in T1 vs T2 [33]. For a small number of participants (n = 6), immune responses could not be measured due to missed visits or their immune response measurements did not pass pre-specified quality control criteria applied by the laboratory prior to unblinding, yielding analyzable immune responses from 94 participants. One participant in the placebo arm and two participants in the vaccine arm deviated from the protocol and were excluded, yielding 18 placebo recipients and 73 vaccine recipients in the current analysis.

HVTN 100 was a phase 1/2 trial testing the safety and immunogenicity of the P_C_/MF59 vaccine regimen [26]. The trial recruited 252 participants who were randomly assigned to P_C_/MF59 (n = 210) or placebo (n = 42). Serum samples and PBMCs were collected two weeks after the fourth vaccination (Month 6.5) and used for immunogenicity measurements. For n = 21 participants, immune responses could not be measured due to missed visits or their immune response measurements did not pass pre-specified quality control criteria applied by the laboratory prior to unblinding, yielding analyzable immune responses from 231 participants. After excluding nine participants for protocol deviations, data from 185 vaccine recipients and 37 placebo recipients remained for this analysis.

### Ethics statement

For the RV144 trial, written informed consent was obtained from all volunteers, who were required to pass a written test of understanding. The study protocol was reviewed by the ethics committees of the Ministry of Public Health, the Royal Thai Army, Mahidol University, and the Human Subjects Research Review Board of the U.S. Army Medical Research and Materiel Command. The World Health Organization and the Joint United Nations Program on HIV/AIDS and the AIDS Vaccine Research Working Group of the National Institute of Allergy and Infectious Diseases at the National Institutes of Health also independently reviewed and endorsed the study protocol. For the HVTN 097 trial, all participants gave written informed consent. The HVTN 097 study protocol was approved by the University of the Witwatersrand Human Research Ethics Committee for Klerksdorp and Soweto sites and by the University of Cape Town Ethics Committee for the Cape Town site. For the HVTN 100 trial, all participants gave written informed consent in English or their local language (Setswana, Sotho, Xhosa, or Zulu). The HVTN 100 study protocol was approved by the research ethics committees of the University of the Witwatersrand, the University of Cape Town, the University of KwaZulu-Natal, and the Medical Research Council.

### Laboratory methods

ICS and BAMA assays were performed blinded to treatment group with validated methods. ICS measurements included CD4+ and CD8+ T-cell responses to vaccine-matched antigens, while BAMA measurements assessed isotype and antigen-specific antibodies to gp140, gp120, vaccine-matched V1V2 antigens and non-matched V1V2 antigens (representing other circulating viruses) [13, 14, 17, 32, 34].

### HIV-1 specific CD4+ and CD8+ T-cell responses

HIV-1-specific CD4+ and CD8+ T-cell responses were assessed by ICS of cryopreserved PBMCs as previously described [13, 16, 26, 35]. No replicates were performed with participant samples due to limited PBMC availability. Responses were evaluated to vaccine-matched antigens (peptides representing gp120 ZM96, 1086.C and TV.1) at the putative peak time point (2 weeks post fourth vaccination). Antigen-specific T-cell subsets were analyzed by COMPASS [16].

### HIV-1 binding antibody responses measured by BAMA

HIV-1 specific IgG (1/50 dilution) and IgG3 (1/40 dilution) for specific vaccine-matched and vaccine-mismatched antigens were measured with serum (HVTN 097) and plasma (RV144, HVTN 100) samples collected at baseline and at the putative peak time point, as previously described [13, 14, 17, 36]. Each sample was tested in duplicate and met preset acceptance criteria before reporting.

### Vaccine-matched responses

Composite variables were created to represent the responses of participants in each study to the vaccine-matched antigen. For example, the composite vaccine-matched gp120-specific CD4+ T-cell polyfunctionality score variable included ALVAC insert-matched responses of RV144 and HVTN 097 participants to the 92TH023 peptides and responses of HVTN 100 participants to the ZM96 peptides. When multiple vaccine-matched responses were measured the maximum magnitude response was used in the composite variable (e.g. gp120 IgG using A244 and MN antigens were both matched to the P_C_/alum regimen). In total, there were 16 vaccine-matched immune responses: 1) Env CD4+ CD154+, 2) Env CD4+ Functionality Score, 3) Env CD4+ IFN-γ\IL-2\CD154+, 4) Env CD4+ IFN-γ+, 5) Env CD4+ IL-2/IFN-γ, 6) Env CD4+ IL-2+, 7) Env CD4+ Polyfunctionality Score, 8) Env CD8+ CD154+, 9) Env CD8+ IFN-γ\IL-2\CD154+, 10) Env CD8+ IFN-γ+, 11) Env CD8+ IL-2/IFN-γ, 12) Env CD8+ IL-2+, 13) IgG gp120 50 Serum, 14) IgG V1V2 50 Serum, 15) IgG3 gp120 40 Serum, and 16) IgG3 V1V2 40 Serum. Seven CD4+ and five CD8+ T cell response variables were highly correlated, therefore Env CD4+ IFN-γ\IL-2\CD154+ and Env CD8+ IFN-γ\IL-2\CD154+ were chosen to represent their respective groups of responses.

### Statistical methods

#### Integration

Validated ICS and BAMA assays were used to assess cellular and humoral responses across all three trials. However, the specific immune responses assessed differed somewhat across the trials, resulting in a total of 64 shared responses out of the 307 immune responses assessed in at least one trial. For the unsupervised clustering analysis, all three data sets were integrated before normalization and transformation (see below), leaving unmeasured responses as missing values. We made the assumption that immune measurements were missing completely at random (MCAR). This is justified since the “missingness” of responses was the result of missed visits and assay quality issues, which are presumably not related to the missing values themselves, nor their missingness. Therefore, the BDPT transformation, under the complete case analysis, would yield appropriate transformed values without biases.

#### Normalization

ICS measurements correspond to percentages of T cells producing specific cytokines upon antigen stimulation. ICS readouts are typically expressed in values ranging from 0 to 100. When internal control readouts are subtracted from experimental readouts, negative percentages are occasionally recorded when the experimental readouts are relatively small. In contrast, BAMA detects the amount of antibody bound to an immobilized antigen. Readout values for this assay range, on a logarithmic scale, from 0 to a positive value. To express ICS and BAMA readout values on a comparable scale, we normalized all measurements on the integrated data set by setting the 95^th^ percentile of each assay readout to one, i.e., all observed values of each immune response assay were divided by its respective 95^th^ percentile value.

#### Bimodal distribution

Immune responses generally have a bimodal distribution wherein the first peak corresponds to non-responders and the second peak corresponds to responders. To examine the distribution of responses across participants for each immune response measurement, we used nonparametric kernel-based density estimation to determine the distribution density for each immune response [37]. In the context of a vaccine trial, the first peak typically includes observed immune response values from placebo recipients and non-responding vaccine recipients (or vaccine recipients with protocol deviation). The second peak, if present, corresponds to immune response values from responder vaccine recipients.

#### Bi-directional power transformation

In addition to their bimodality nature, immune responses among responders tend to have a wide spread, often resulting in a highly right-tail skewed distribution from zero to a large positive value. This wide spread hinders clear identification of the two peaks corresponding to non-responders and responders. To overcome this interpretation hurdle, we used a bi-directional power transformation (BDPT) to transform individual immune responses so that the mixed distributions are converted into clearly bimodal distributions, improving analysis and visualization of non-responders and responders.

As an extension to the power transformation and the Box-Cox transformation [38], BDPT applies power transformations on observed values below a threshold value and on observed values above the same threshold value via

$$Z=\{\begin{array}{ccc} -\omega {\left[(\theta -Y)/m_{\mathrm{negative}}\right]}^{\rho } & & Y<\theta \\ \varpi {\left[(Y-\theta )/m_{\mathrm{positive}}\right]}^{\delta } & & Y\geq \theta \end{array},$$

where (*ω,ρ*) and (*ϖ,δ*), respectively, are a pair of scale and power parameters for those values below and above the threshold value, and (*m*_negative_,*m*_positive_) are targeted median values for the two separate peaks. Both the scale parameters (*ω,ϖ*) and power parameters (*ρ,δ*) are treated as tuning parameters. The scale parameters dictate centralities of transformed values for non-responders and responders, while the power parameters influence symmetricities of transformed values for non-responders and responders. The choice of these parameters influences the transformed immune response values and hence their associated interpretations. The two scale parameters (*ω,ϖ*) are chosen so that the transformed immune response values have interpretable intervals, e.g., -2 to 0 for non-responders’ values and 0 to a number around 10 for responders’ immune response values. Note some transformed positive values can be greater than 10, but are truncated in the heatmaps, to enable visual interpretation of the heatmaps. Given the relatively small sample sizes in three vaccine trials considered here, the power parameters (*ρ,δ*) were set to (0.25, 0.25) and the scale parameters (*ω,ϖ*) were set to (1, 5), so that their empirical distributions for “non-responders” and “responders” appeared to be symmetric, and median values for respective groups equal approximately 1 and 5. Note that transformed values were used for all analyses, unless noted otherwise.

To provide an intuitive motivation for BDPT, we show how mixing responders’ and non-responders’ measurements induced a skewed bimodal distribution (Panels A, B and C in S1 Fig), and how BDPT can be used to transform the measurements so that the mixture right-tail skewed distribution is turned into a “more interpretable” bimodal distribution, potentially improving visual display and result interpretation (Panel D in S1 Fig).

#### Hierarchical clustering and visualization

We compute, separately, the Euclidean distance of immune response values between participants and between immune responses, and then apply the hierarchically clustering methods, separately clustering subjects and clustering immune responses. The basic idea of the hierarchical clustering is to place columns (or rows) closer if their distances are relatively small. We used the Ward method [39], i.e. the sum of the squared distances is used as an objective function and thus a pair of columns (rows) with the smallest objective function value would be organized closer together. Participants with similar patterns of immune responses are clustered more closely to one another, while participants with more divergent patterns of immune responses are placed into different row clusters. Likewise, immune responses similar across participants are clustered more closely to one another, whereas immune responses with more divergent patterns across participants are placed into different column clusters. This analytic and visualization strategy gains substantial tractions for unsupervised learning in high dimensional data analyses [29].

The hierarchical clustering algorithm is applicable to BDPT transformed immune response values, as well as to other transformed values. As sensitivity analysis, we clustered “dichotomized immune responses” and “log-transformed immune response” by the same algorithm and obtained comparable cluster patterns (not shown) to those presented here, although their numerical properties and interpretabilities are different.

#### Dynamic ranges of immune responses

One important factor determining the statistical power of assessing association of vaccine-induced immune response with risk of HIV infection (or protection against HIV infection) is the biologically relevant dynamic range of the variable [e.g., see power calculations in [40]]. We calculate two measures of dynamic range for all 64 immune response variables: 1) the interquartile range, i.e., the range between the 25^th^ and 75^th^ percentiles; and 2) the range between the 10^th^ and 90^th^ percentiles. Dynamic ranges may be validly compared among immune response variables measured by the same assay (i.e., within BAMA or within ICS), but not between assays. For dynamic range calculations, BDPT-transformed scales were used.

#### Adjusted Spearman’s correlation

When many immune responses are measured, it is of interest to identify highly correlated (i.e. redundant) responses to improve cost-effectiveness. To this end, we compute pairwise correlation coefficients between all immune responses within each Fig 1 immune response cluster, excluding the CD8+ T-cell response cluster, which lacked sufficient variability. To adjust covariate-induced heterogeneity in the evaluation of nonparametric correlations between immune response measurements, rank-based Spearman correlation coefficients are computed, after adjusting pertinent covariates, using the methods developed by Shepherd and colleagues [41–43].

#### Statistical software tools

All analyses were performed in R (https://www.r-project.org/). For hierarchical clustering, the function hclust was used with customized distance function and related options. Clustered results were displayed the heatmap.3 package, the radarchart function was used to display immune response patterns among participant clusters, and the mosaic function was used to visually display the contingency table. Chi-square test statistics for assessing associations between two categorical variables were calculated using the chisq.test function. In this function, the asymptotic chi-square distribution is used to compute p-values or the bootstrap method is used to compute p-values when some frequencies are too small for accurate asymptotic approximation calculation. All computations were implemented in the R language.

#### MetaVis

Row metadata (subject covariates) and column metadata (immune response annotations) can be explored using the interactive tool MetaVis (http://sieve.fhcrc.org/metavis_RV144_097_100).

## Supporting information

S1 Fig Illustration of bidirectional power transformation (BDPT). A) Distribution of immune response levels among non-responders, B) Distribution of immune response levels among responders, C) Mixture distribution of immune response levels among both responders and non-responders, and D) Distribution of BDPT-transformed response levels, showing a mixture of responders and non-responders.
(PDF)

S2 Fig Heatmap representations of immune responses observed in the three trials. A) Heatmap of the 307 immune responses observed in 538 participants, in which white spaces indicate uncollected values; B) Heatmap of 153 immune responses in 225 participants in RV144; C) Heatmap of 164 immune responses in 91 participants in HVTN 097; and D) Heatmap of 198 immune responses in 222 participants in HVTN 100.
(PDF)

S1 Table List of the 64 immune responses shared by the RV144, HVTN 097, and HVTN 100 studies. (XLSX)

S2 Table Distributional statistics of the 12 immune responses for which vaccine recipients consistently showed limited or no response. Left panel, original values; right panel, normalized and transformed values.
(XLSX)
