# Supplementary material for: Landscapes of binding antibody and T-cell responses to pox-protein HIV vaccines in Thais and South Africans
Source: PLoS One. 2020 Jan 30;15(1):e0226803. doi: 10.1371/journal.pone.0226803 (PMC6992005; doi:10.1371/journal.pone.0226803)
Supplement: S1 Fig — A) Distribution of immune response levels among non-responders, B) Distribution of immune response levels among responders, C) Mixture distribution of immune response levels among both responders and non-responders, and D) Distribution of BDPT-transformed response levels, showing a mixture of responders and non-responders. (PDF) [file pone.0226803.s001.pdf]

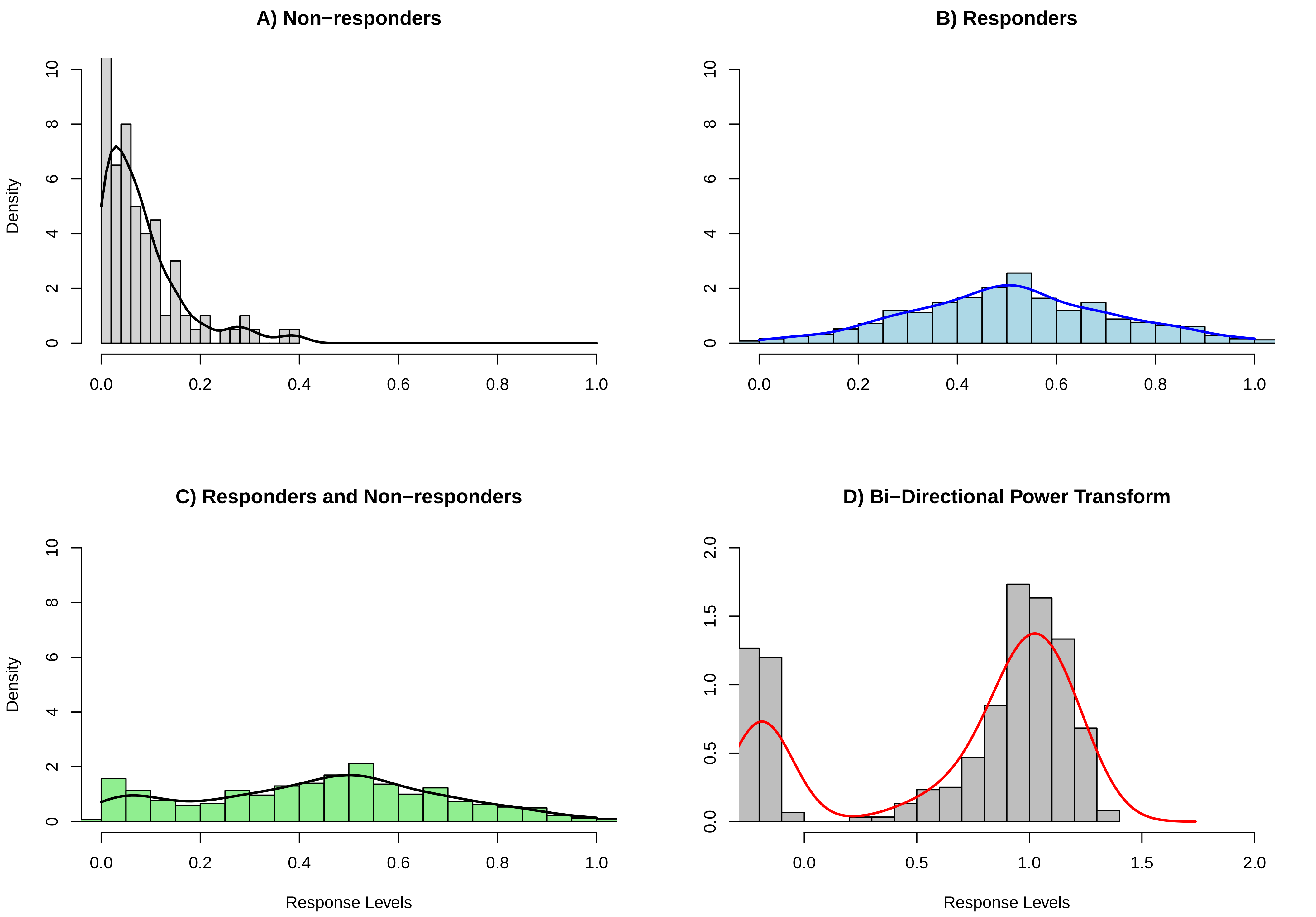

A) Non−responders
B) Responders
C) Responders and Non−responders
D) Bi−Directional Power Transform
Density
Density
Response Levels
Response Levels
0
2
4
6
8
10
0.0
0.2
0.4
0.6
0.8
1.0
0.0
0.5
1.0
1.5
2.0
