# Supplementary material for: Landscapes of binding antibody and T-cell responses to pox-protein HIV vaccines in Thais and South Africans
Source: PLoS One. 2020 Jan 30;15(1):e0226803. doi: 10.1371/journal.pone.0226803 (PMC6992005; doi:10.1371/journal.pone.0226803)
Supplement: S2 Fig — A) Heatmap of the 307 immune responses observed in 538 participants, in which white spaces indicate uncollected values; B) Heatmap of 153 immune responses in 225 participants in RV144; C) Heatmap of 164 immune responses in 91 participants in HVTN 097; and D) Heatmap of 198 immune responses in 222 participants in HVTN 100. (PDF) [file pone.0226803.s002.pdf]

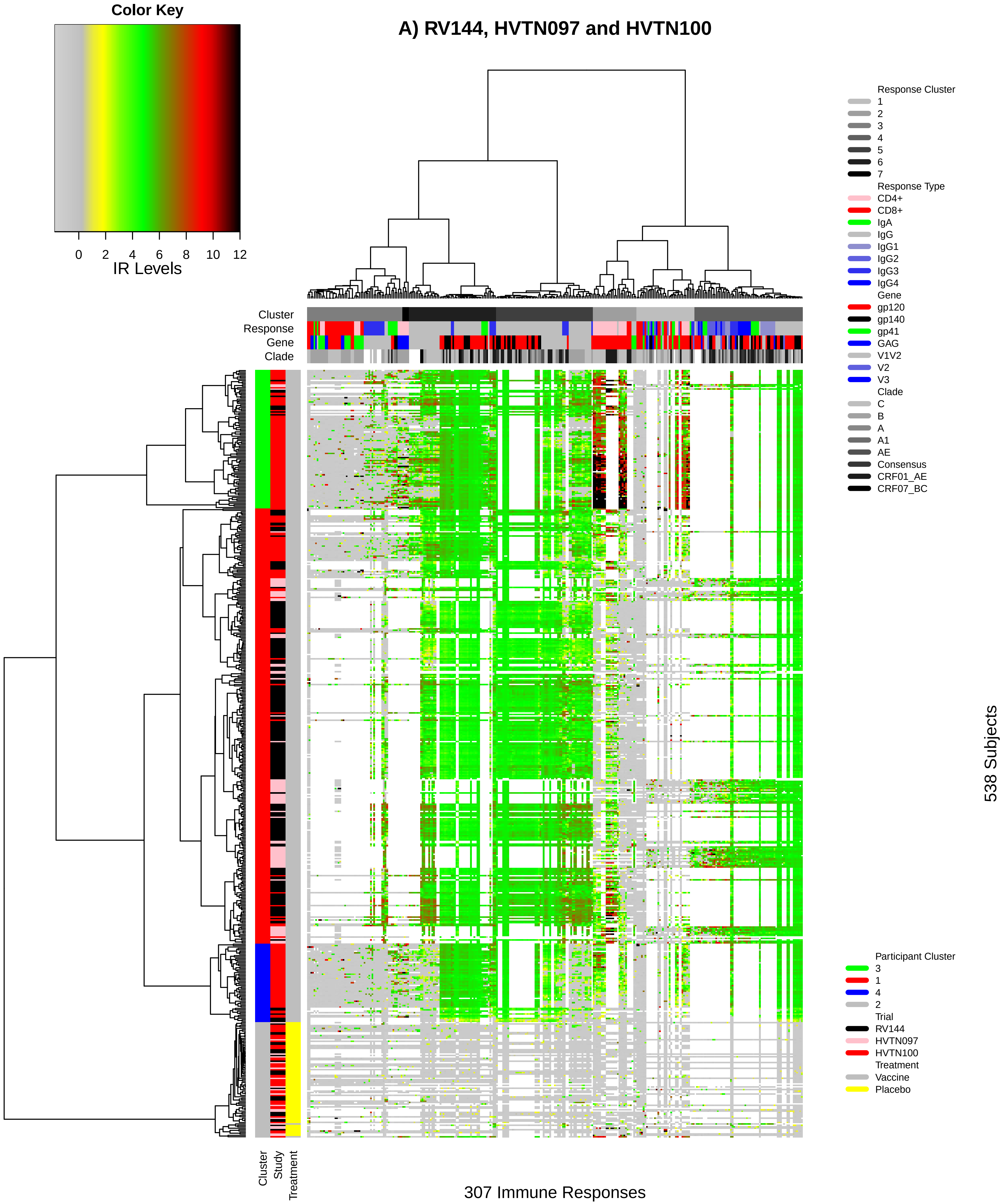
Color Key
0 2 4 6 8 10 12
IR Levels
A) RV144, HVTN097 and HVTN100
Response Cluster
1
2
3
4
5
6
7
Response Type
CD4+
CD8+
IgA
IgG
IgG1
IgG2
IgG3
IgG4
Gene
gp120
gp140
gp41
GAG
V1V2
V2
V3
Clade
C
B
A
A1
AE
Consensus
CRF01_AE
CRF07_BC
Cluster
Response
Gene
Clade
Participant Cluster
3
1
4
2
Trial
RV144
HVTN097
HVTN100
Treatment
Vaccine
Placebo
538 Subjects
Cluster
Study
Treatment
307 Immune Responses

# B) RV144

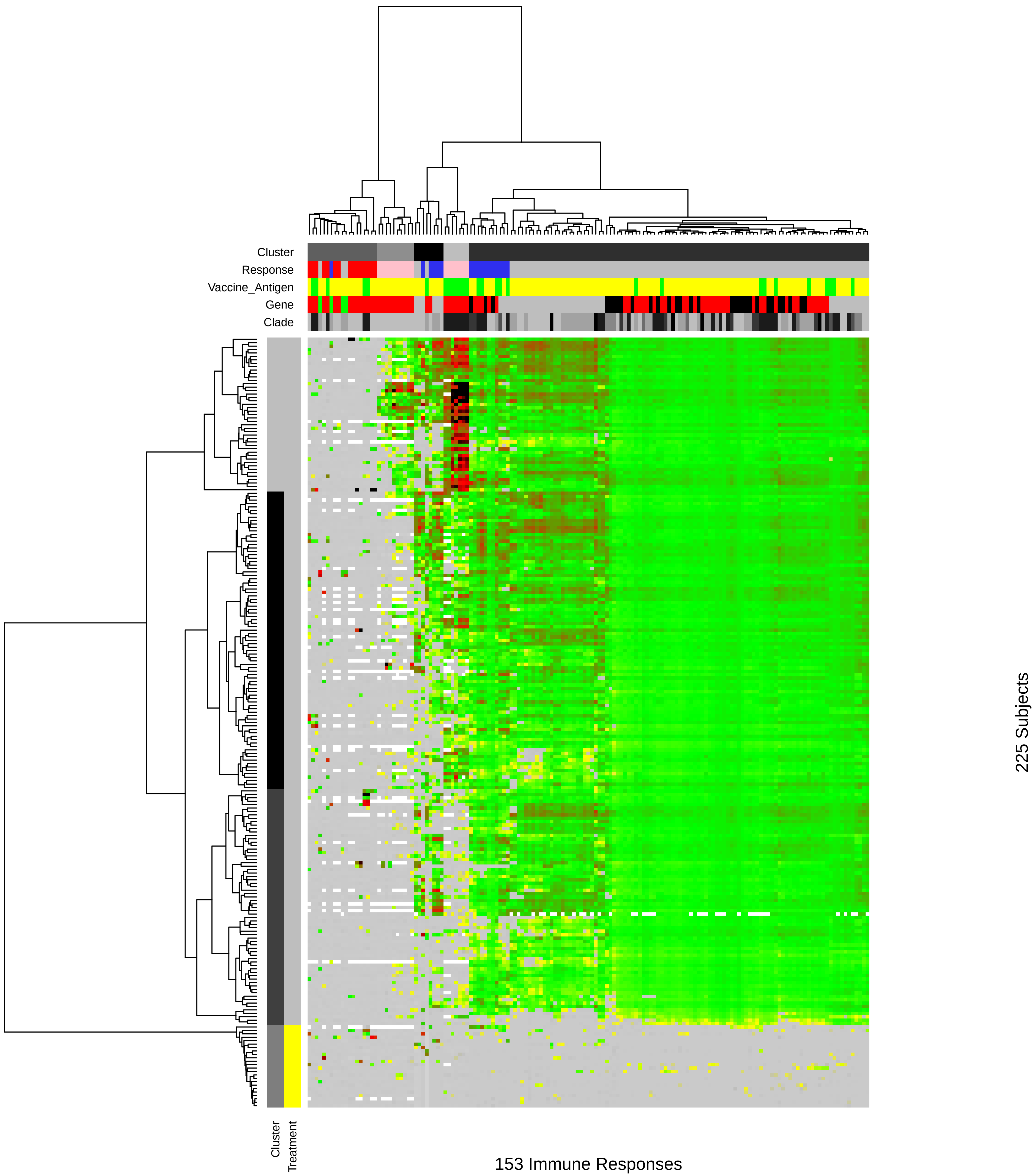
Cluster
Response
Vaccine_Antigen
Gene
Clade
225 Subjects
Cluster
Treatment
153 Immune Responses

c) HVTN097

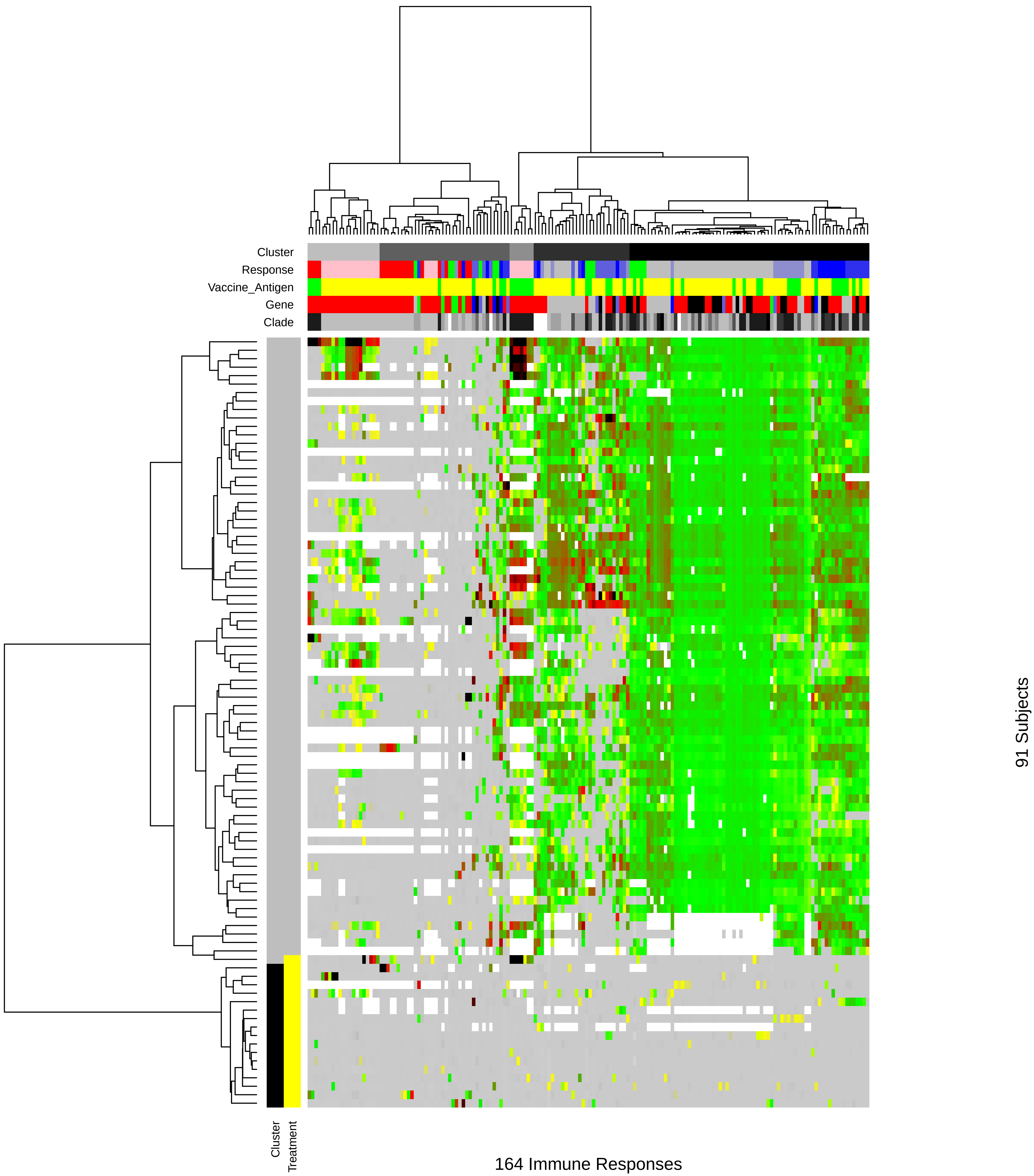
Cluster
Response
Vaccine_Antigen
Gene
Clade
91 Subjects
Cluster
Treatment
164 Immune Responses

## D) HVTN100

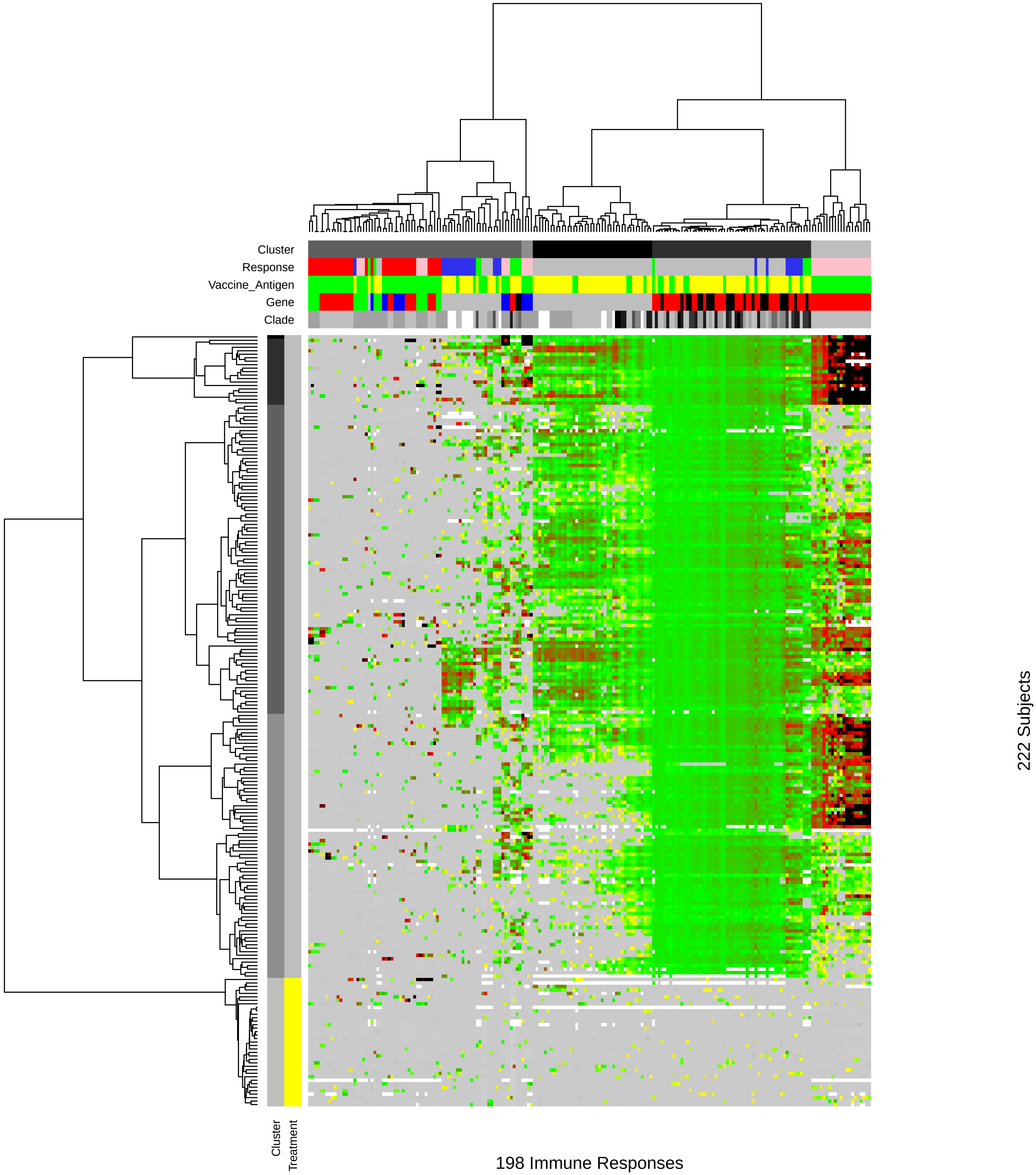

Cluster
Response
Vaccine_Antigen
Gene
Clade
Cluster
Treatment
198 Immune Responses
222 Subjects
